# Preclinical characterization of MTX-101: a novel bispecific CD8 Treg modulator that restores CD8 Treg functions to suppress pathogenic T cells in autoimmune diseases

**DOI:** 10.3389/fimmu.2024.1452537

**Published:** 2024-11-04

**Authors:** Jennifer L. Gardell, Meghan E. Maurer, Monica M. Childs, Minh N. Pham, Brent Meengs, Susan H. Julien, Cong Tan, Daniel R. Boster, Phoenicia Quach, Jon H. Therriault, Gleda Hermansky, Daniel T. Patton, Justin Bowser, Alex Chen, Nadine N. Morgan, Emily A. Gilbertson, Lisa Bogatzki, Kaelen Encarnacion, Catherine J. McMahan, Courtney A. Crane, Kristine M. Swiderek

**Affiliations:** Mozart Therapeutics, Seattle, WA, United States

**Keywords:** CD8 regulatory T cells, immunomodulatory bispecific antibodies, autoreactive CD4 T cells, autoimmune disease affected tissue organoids, killer immunoglobulin like receptor (KIR)

## Abstract

**Introduction:**

Regulatory CD8 T cells (CD8 Treg) are responsible for the selective killing of self-reactive and pathogenic CD4 T cells. In autoimmune disease, CD8 Treg may accumulate in the peripheral blood but fail to control the expansion of pathogenic CD4 T cells that subsequently cause tissue destruction. This CD8 Treg dysfunction is due in part to the expression of inhibitory killer immunoglobulin-like receptors (KIR; KIR2DL isoforms [KIR2DL1, KIR2DL2, and KIR2DL3]); these molecules serve as autoimmune checkpoints and limit CD8 Treg activation.

**Methods:**

Here we describe the pre-clinical characterization of MTX-101, a bispecific antibody targeting inhibitory KIR and CD8. Using human peripheral blood mononuculear cells (PBMC) derived from healthy donors and autoimmune patients, humanized mouse models, and human derived tissue organoids, we evaluated the molecular mechanisms and functional effects of MTX-101.

**Results:**

By binding to KIR, MTX-101 inhibited KIR signaling that can restore CD8 Treg ability to eliminate pathogenic CD4 T cells. MTX-101 bound and activated CD8 Treg in human peripheral blood mononuclear cells (PBMC), resulting in increased CD8 Treg cytolytic capacity, activation, and prevalence. Enhancing CD8 Treg function with MTX-101 reduced pathogenic CD4 T cell expansion and inflammation, without increasing pro-inflammatory cytokines or activating immune cells that express either target alone. MTX-101 reduced antigen induced epithelial cell death in disease affected tissues, including in tissue biopsies from individuals with autoimmune disease (i.e., celiac disease, Crohn’s disease). The effects of MTX-101 were specific to autoreactive CD4 T cells and did not suppress responses to viral and bacterial antigens. In a human PBMC engrafted Graft versus Host Disease (GvHD) mouse model of acute inflammation, MTX-101 bound CD8 Treg and delayed onset of disease. MTX-101 induced dose dependent binding, increased prevalence and cytolytic capacity of CD8 Treg, as well as increased CD4 T cell death. MTX-101 selectively bound CD8 Treg without unwanted immune cell activation or increase of pro-inflammatory serum cytokines and exhibited an antibody-like half-life in pharmacokinetic and exploratory tolerability studies performed using IL-15 transgenic humanized mice with engrafted human lymphocytes, including CD8 Treg at physiologic ratios.

**Conclusion:**

Collectively, these data support the development of MTX-101 for the treatment of autoimmune diseases.

## 1 Introduction

In autoimmune diseases, the immune system lacks the ability to distinguish “self” from “non-self” proteins, and select antigens can initiate a proinflammatory immune response that expands CD4 T cells that cause autoimmune pathology. Although the exact causes of autoimmune diseases are not completely understood, microbial or viral insults in combination with genetic predisposition and additional environmental factors contribute to the onset of autoimmune diseases or trigger disease flares (1, 2). In the context of an antimicrobial or antiviral immune response, expansion of CD4 T cells specific for the pathogen may cross-react with self-antigens, which may be retained during thymic selection to support antiviral and antimicrobial responses. If left unrestrained, self-cross-reactive CD4 T cells can become pathogenic and attack host-derived tissues. Regulatory T cells (Tregs; e.g., CD4 Tregs and CD8 Tregs) suppress inflammatory responses and are important for maintaining homeostasis and tolerance to self-antigens. Specifically, CD8 Tregs are a population of cytotoxic CD8 T cells with oligoclonal T-cell receptors (TCRs (3)) that are specific for potentially pathogenic CD4 T cells (1).

Over the last 50 years, several studies have described a subpopulation of CD8 Tregs with an ameliorating effect on inflammatory responses in murine models of disease (4–8) as well as in human autoimmune diseases (9–12). CD8 Tregs are distinct from, and likely work in concert with, CD4 Tregs in both primary mechanisms of action and impact on inflammatory cascade pathology. CD4 Tregs primarily function during active inflammation through a variety of described mechanisms, including the production of anti-inflammatory cytokines and adenosine, depletion of IL-2, and tolerization of antigen-presenting cells (13). CD8 Tregs can act upstream of the autoimmune inflammatory cascade, preventing pathogenic T-cell expansion and consequently reducing self-reactive antibody production and proinflammatory cytokines in the periphery and tissue (1). Therefore, in autoimmune disease, the elimination of pathogenic cells prior to multi-cellular and robust inflammation may have a more durable impact on the establishment and maintenance of immune balance.

Recent data have defined a CD8 Treg phenotypic signature that is conserved in healthy individuals and individuals with autoimmune disease. CD8 Tregs display an expression profile of effector cytotoxic cells and require direct cell-to-cell contact, leading to the elimination of pathogenic CD4 T cells (8). Consistent with this observation, the increased prevalence of functional CD8 Tregs is associated with the delayed onset of type I diabetes (T1D) in at-risk populations (9, 10) and improved outcomes in individuals with multiple sclerosis (MS) (11). Despite increased CD8 Treg numbers in populations with CD4-driven autoimmune diseases, the expansion of pathogenic CD4 T cells is not sufficiently inhibited to prevent pathology and/or disease progression. The lack of productive engagement with pathogenic CD4 T cells may be due partly to the presence of inhibitory receptors on the CD8 Treg surface that regulate their threshold for activation, including inhibitory KIRs, which engage major histocompatibility complex (MHC) class I on target cells (14). Inhibitory KIRs compete with the TCRs for MHC class I/peptide binding and send inhibitory signals that counteract activating stimuli (15). We postulate that restoration of TCR/MHC/peptide interactions and blocking of KIR inhibitory signaling would restore the elimination of pathogenic CD4 T cells via functional CD8 Tregs. We have developed a bispecific antibody (MTX-101) that targets both CD8 and the inhibitory immune checkpoint KIR2DL, which are co-expressed on the surface of CD8 Tregs. MTX-101 was engineered to include a blocking Fab binding domain directed to inhibitory KIRs (16) and a non-blocking, non-signaling scFv binding domain directed to CD8 (17) to promote interactions with cells expressing both inhibitory KIRs and CD8.

In this study, we evaluated the binding, pharmacokinetics, non-clinical safety, mechanism of action, and effects of MTX-101 on CD8 Tregs. *In vitro* and *in vivo* testing showed that the selective activation of human CD8 Tregs using MTX-101 reduced inflammation without broad immunosuppression or unwanted immune cell activation across the translational systems evaluated. Collectively, our data support MTX-101 as a novel and selective approach to restoring CD8 Treg network functions in the setting of CD4 T cell-driven autoimmune disease.

## 2 Materials and methods

### 2.1 Surface staining of human donor PBMCs

Peripheral blood mononuclear cells (PBMCs) from healthy donors (Bloodworks Northwest, Seattle, WA, USA; StemCell Technologies, Vancouver, BC, Canada) were thawed and rested overnight at 37°C in human T-cell media (huTCM; X-VIVO^®^ 15, 5% human AB serum, 1× penicillin/streptomycin, 1× GlutaMax™ supplement). Cells were then plated at 3e5 cells/well in 96-well plates and stimulated with or without 1 µg/mL anti-CD3 (OKT3, BioLegend, San Diego, CA, USA) for 24 hours before staining for phenotypic markers. Antibodies against KIR2DL1, KIR2DL2/3, and KIR3DL1 were allophycocyanin-labeled, and together, that population was considered “KIR+”. Prior to staining, the cells were treated with Fc block and Zombie Aqua™ (BioLegend) for 15 minutes at room temperature. Extracellular staining was performed at 4°C for 20 minutes before fixing and permeabilizing the cells. Intracellular staining was performed using the eBioscience™ Intracellular Fixation and Permeabilization Buffer Set (Thermo Fisher, Waltham, MA, USA), and intranuclear staining was performed using the True-Nuclear™ Transcription Factor Buffer Set (BioLegend). Cells were analyzed on a FACSymphony™ A1 cell analyzer (BD Biosciences, San Jose, CA, USA), and data were analyzed using FlowJo™ software (version 10.5 or later, BD).

### 2.2 Restimulation assay

PBMCs derived from donors with celiac disease (Sanguine Biosciences, Woburn, MA, USA) were plated at 5e6 cells/mL in human T-cell media (huTCM; X-VIVO^®^ 15 with 5% human AB serum, 1× GlutaMax™ supplement and 1× penicillin/streptomycin) in 6-well plates with gliadin peptides (**Supplementary Table 1**, Elim BioPharmaceuticals, San Francisco, CA, USA; 12.5 µg/mL of each of seven peptides). huTCM containing 5 ng/mL each of IL-2 and IL-15 was added 4 days post-peptide addition and every 3–4 days thereafter for 3 weeks. On day 21, autologous PBMCs were thawed, and antigen-presenting cells (APCs; CD3^+^- and CD56^+^-depleted cell subsets, StemCell Easy Sep™ Human CD3 Positive Selection Kit II, cat# 17851, and Human CD56 Positive Selection Kit II, cat# 17855) were plated at 50,000 cells/well and stimulated with 5 µg/mL of each of seven gliadin peptides, 0.02 µg/mL anti-CD3 (OKT3) antibody, or media alone. On day 22, expanded PBMCs were sorted into CD5+CD4+IntB7+ and CD5+CD4−KIR2D+ populations using a FACSAria™ Fusion flow cytometer (BD Biosciences). IntB7+CD4+ targets (100,000 cells/well) and KIR2D+CD8+ Tregs (20,000 cells/well) were added to 96-well plates with autologous stimulated APCs. Cultures were treated with 100 nM MTX-101 or media alone. Supernatants were collected at 3 days post-restimulation for cytokine analysis, and cells were stained on day 3 for flow cytometry.

### 2.3 Incucyte assay cell preparation and killing assay

To generate effector cells, PBMCs derived from a donor with celiac disease (Sanguine Biosciences) were thawed and plated in huTCM with 5 ng/mL IL-7 (BioLegend) and 2.5 ng/mL IL-15 (BioLegend) for 7 days, including a complete change in media and addition of cytokines on day 2 and day 5. On day 7, cells were harvested, stained, and flow-sorted using the following strategy: CD5+/NKp46−/CD4−/CCR7−/CD28−. An aliquot of cells was stained for CD8 and KIR to determine the percentage of CD8+KIR+ cells resulting from sorting and used to calculate the number of CD8 Tregs present at each effector-to-target ratio. GFP+ SKW CD4 cells engineered to express a TCR responsive to gliadin peptide alpha-1a (NPL001, Elim BioPharmaceuticals) (GFP+ SKW-LS2.8, a kind gift of ImmunsanT, Cambridge, MA, USA) or parental SKWs (pSKWs) were stimulated using CD14+ cells isolated from PBMCs derived from a healthy donor (Bloodworks Northwest) with an HLA-DQ2.5 haplotype. PBMCs were plated in huTCM with 10 µg/mL NPL001 and 5 ng/mL IFN-γ (BioLegend). Unactivated targets did not receive NPL001 or IFN-γ stimulation. Equal numbers of SKW-LS2.8 or pSKW cells were plated on top of CD14 cells and allowed to incubate overnight prior to use in the assay. Activated or unactivated SKW-LS2.8 or pSKW target cells were plated in poly-l-lysine coated 384-well, optically clear plates at 20,000 cells/well in 50 µL huTCM and allowed to settle for 30–60 minutes at room temperature before being placed in an incubator. Effector cells were plated on top in 50 µL at various ratios to achieve the number of CD8 Tregs per well as calculated using %KIR+CD8+ in the sorted population. The plate was read in the IncuCyte^®^ cell analysis system (Sartorius, Ann Arbor, MI, USA) every 4 hours for green fluorescent protein (GFP) signal. The percent change in GFP+ objects from the 8-hour timepoint was graphed.

### 2.4 Binding affinity by Octet^®^

The binding affinity of MTX-101 to human KIRs and human CD8α was determined by Bio-Layer Interferometry utilizing an Octet^®^ Red 384 instrument (Sartorius). After MTX-101 was captured onto an anti-human IgG Fc Capture (AHC) sensor tip, a dilution series of the monomeric His-tagged target analyte [KIR2DL1 (Sino Biological, cat# 13145-H08H, Wayne, PA, USA), KIR2DL2 (in-house product), KIR2DL3 (Sino Biological, cat# 12828-H08H), or CD8α (Sino Biological, cat# 10980-H08S)] was allowed to bind to determine affinity. Ten minutes of association followed by 15 minutes of dissociation was utilized for these measurements. Co-binding experiments were performed by coupling KIR2DL2-Fc (R&D Systems, Minneapolis, MN, USA, cat# 3015-KR-050) and CD8α to separate AR2G (amine-reactive) sensor tips (Sartorius) via amine chemistry (EDC/NHS). MTX-101 was allowed to bind to the immobilized antigen, followed by further binding of antigen (either KIR2DL2-Fc or CD8α) to the captured bispecific molecule. Simultaneous co-binding of both antigens to MTX-101 was verified with an increase in mass on the sensor surface. Species cross-reactivity experiments were performed by capturing His-tagged KIRs (human KIR2DL1, human KIR2DL3, cynomolgus KIR1DL, cynomolgus KIR2DL04, cynomolgus KIR3DL07, and murine Ly49F) and His-tagged CD8α [human CD8α and murine CD8α (Sino Biological, cat# 50389-M08H)] onto Ni-NTA (nickel-charged tris-nitriloacetic acid) sensor tips (Sartorius). Cynomolgus CD8α-Fc (Sino Biological, cat# 90888-C02H) was biotinylated and captured onto a SA (streptavidin) sensor tip (Sartorius). Commercial sources for cynomolgus KIRs and mouse Ly49F were not available. For these reagents/analytes, plasmids containing the predicted extracellular domain followed by a C-terminal 6xHis tag were transfected and expressed in-house from the Expi293 transient expression system and purified. Ten minutes of association followed by 10 minutes of dissociation was utilized for these measurements. Buffer reference subtraction was applied to all binding curves. Association and dissociation rate constants were globally fit to a 1:1 Langmuir binding model, and K_D_ binding affinity was calculated using Octet Analysis Studio (Sartorius).

### 2.5 Generation of cell lines stably expressing the target protein

SKW cells were resuspended at 1 × 10^6^ cells per mL in pre-warmed target media. Lentiviral particles for CD8 and KIR2DL1 (Origene, Rockville, MD, USA) were added according to the desired multiplicity of infection (MOI) along with polybrene (8 µg/mL; Sigma-Aldrich, St. Louis, MO, USA) in a 1.5-mL microcentrifuge tube. Lentivirus cells were incubated for 20 minutes at room temperature prior to spinoculation for 30 minutes at 800× gravity at 32°C. Following centrifugation, virus-containing cells were incubated for 48 hours at 37°C in a 48-well plate and expanded under antibiotic selection to generate a polyclonal stable cell line.

### 2.6 On-cell and PBMC binding

After thawing, transduced cells or donor PBMCs were harvested, and the cell count was determined based on the viability measured using the Countess™ cell counter (Thermo Fisher). Cells were stained with live/dead Zombie viability dye (Thermo Fisher) for 20 minutes at room temperature and then plated at 2e5 cells/well in a 96-well plate for drug binding. The cells were incubated with serial dilution of MTX-101 for 30 minutes on ice in the dark. Cells were then incubated with anti-human Fc secondary antibody (Jackson Immunoresearch Laboratories, West Grove, PA) for 30 minutes at 4°C in the dark followed by staining for extracellular markers. Cells were then fixed with intracellular fixative for 20 minutes at room temperature and analyzed on a FACSymphony™ A1 cell analyzer (BD Biosciences).

### 2.7 Transfection of Expi293 cells for off-target binding assay

Fifty mL of Expi293 cells (Thermo Fisher) was cultured to a density of 1.5e6 cells/mL in 125-mL flasks. Cells were allowed to grow overnight at 37°C, 8% CO_2_, and 150 RPM in Expi293 Expression Medium (Thermo Fisher). The following day, the cells were counted (between 2.7 to 3e6 cells/mL, > 90% viable) and transfected using 1 mg/mL polyethyleneimine (PEI; Polysciences, Warrington, PA, USA) as the complexing reagent at a concentration of 3.5 mg/L with a DNA concentration of 1 mg/L. Then, 16.66 µg of each plasmid was combined with 175 µL of 1 mg/mL PEI using 2.5 mL Opti-MEM™ Reduced Serum Medium (Thermo Scientific) for both PEI and DNA. These solutions were then combined to make 5 mL DNA/PEI complexes. The DNA/PEI mixtures were incubated for 30 minutes at room temperature with periodic mixing. Cells were then incubated with PEI/DNA complexes at 37°C. Eighteen hours after transfection, cells were fed with 2.5 mL CHO Efficient Feed B Nutrient Supplement (Thermo Scientific, cat# A1024001) and 400 µL 500 mM valproic acid (Fisher, cat.# 501786601) to aid transfection efficiency. The cells were grown in the Kuhner Climo-Shaker ISF1-XC Incubator at 150 RPM, 37°C, and 8% CO_2_ for another 48 hours prior to performing a binding assay with MTX-101 or KIR3DL1 targeted antibodies.

### 2.7 KIR and CD8 receptor quantification

KIRs and CD8 receptors were quantified on CD8 Tregs from PBMCs from healthy donors or donors with celiac or Crohn’s disease using quantification beads (Quantum Simply Cellular, Bangs Laboratories Inc., Fishers, IN, USA, cat# 815). Four sets of beads with known numbers of anti-mouse IgG binding sites were used to generate a standard binding curve that was used to calculate antibody binding sites per individual cell. ​Quantification beads and samples were mixed together and stained following standard procedures and then analyzed on a FACSymphony™ A1 cell analyzer (BD Biosciences).​ KIRs and CD8 geometric mean fluorescence intensity (gMFI) were evaluated for each antibody and used for the calculation of KIRs and CD8 antibody binding sites on CD8 Tregs.

### 2.8 Bound KIR receptor measurement

PBMCs from healthy donors (Bloodworks NW) were thawed and rested overnight in huTCM in 50-mL conical tubes​. CD3 T cells were isolated from rested PBMCs (StemCell negative selection kit​, cat# 17951). 2e5 T cells were plated per well, and a dose titration of MTX-101 up to 1,600 nM was incubated for 30 minutes at 4°C. Cells were then washed and incubated with 800 nM of KIR-Fc for 20 minutes at 4°C. Extracellular immune cell phenotyping antibodies were added for an additional 30 minutes at 4°C, and cells were then washed and analyzed on a FACSymphony™ A1 cell analyzer (BD Biosciences).

### 2.9 Crohn's flagellin and OmpC antigen stimulation assay

Frozen PBMCs derived from donors with Crohn’s disease or healthy donors were thawed and rested overnight in huTCM media. Rested cells were counted, labeled with a carboxyfluorescein succinimidyl ester (CFSE; Thermo Fisher), and plated in a 96-well, round-bottom plate at 2e5 cells/well in huTCM media; 100 ng/mL bacterial flagellin (Invivogen, San Diego, CA, USA) (18) and 1 µg/mL OmpC _321-340_ peptide (GenScript, Piscataway, NJ, USA) (19) were added for stimulation. MTX-101 (100 nM) was added to the treated wells. Half-medium changes with the cytokines IL-7 and IL-15 (final concentration 10 ng/mL) were performed on day 3 and day 5 of the PBMC culture. Supernatants collected on day 5 and day 7 were placed at −20°C for future cytokine analysis by Meso Scale Discovery (MSD) U-Plex assay (Meso Scale Diagnostics, Rockville, MD, USA). Cells were harvested and stained for flow cytometry on day 7.

### 2.10 Primary intestinal tissue organoid culturing

Duodenal and colonic tissue was received from celiac and Crohn’s donors (Dr. James Lord laboratory, Benaroya Research Institute) and processed for organoid generation following digestion protocol. The tissue was resuspended in a collagen matrix and divided into six trans-well membranes for organoid generation with supplemental growth factors. The organoids were allowed to establish growth for at least 9 days, and then media was replaced with media including antigenic stimulation (celiac, gliadin peptides and Crohn's, flagellin and OmpC peptides) with or without the addition of MTX-101 for 3 to 4 days. Following incubation, organoids were harvested, collagenase was used to digest the Matrigel, and immune and epithelial cells were stained for analysis by flow cytometry.

### 2.11 Activation of immune cell subsets in PBMC culture+/− anti-CD3

Healthy, celiac, and Crohn’s donor PBMCs were plated at 2 × 10^6^ per well in the presence of increasing doses of MTX-101 with and without additional anti-CD3 (Ultra-LEAF purified anti-human CD3, clone OKT3, BioLegend) stimulation for 24 or 48 hours at concentrations as specified. MTX-101-treated cells were then stained with anti-human Fc antibody (Jackson ImmunoResearch Laboratories) to detect MTX-101 binding to CD8 Tregs or other immune cell populations following the binding protocol procedure as described above. Cells were also stained for surface phenotyping and activation (CD69 and ICOS) markers following incubations.

### 2.12 Immune response to common pathogens

PBMCs from three healthy donors (StemCell Technologies) and three celiac donors (Sanguine Biosciences) were plated at 0.5 × 10^6^ cells/well in 48-well plates and pulsed with 1 µg/mL pooled peptides from JPT Peptide Technologies (Berlin, Germany) (CEFT, Influenza A, SARS-CoV-2, or AAV5/6/8), 5 µg/mL tetanus toxoid (Millipore Sigma, Rockville, MD, USA), 37.5 µg/mL gliadin peptides (12.5 µg/mL of each of three peptides), or dimethyl sulfoxide (Thermo Fisher) alone. Cultures were fed fresh media with 5 ng/mL IL-2 on days 7 and 10 post-peptide addition. On day 13, expanded PBMCs were combined in 96-well plates at a 1:1 ratio with newly thawed autologous PBMCs. Cultures were untreated, restimulated with the same antigen that PBMCs were exposed to during the expansion phase, or stimulated with 100 ng/mL SEB (Fisher Scientific) as a positive control. Additionally, cultures were treated with 10 µg/mL (80.4 nM) MTX-101 or media alone. Supernatants were collected at 5 days post-restimulation for cytokine analysis. Supernatants were diluted at 1:50. IFN-γ levels were analyzed using 96-well single-spot plates from Meso Scale Diagnostics. Standards and samples were run in duplicate following the manufacturer’s instructions with the exception that the standard curve was adjusted to cover a broader range of concentrations (10–153,500 pg/mL IFN-γ). Plates were visualized on the MESO QuickPlex SQ 120 imager, and exported data were analyzed using MSD Discovery Workbench (Meso Scale Diagnostics).

### 2.13 Materials derived from human donors

All human materials (blood, PBMCs, and tissues) were derived from donors, and proper consent had been accredited by organizations for the use of their materials for research purposes at a commercial entity: human blood was purchased from Bloodworks Northwest (IRB Protocol 20141589) or prospectively collected under contract with Sanguine Biosciences Inc. (IRB tracking number 20242074, 20224886) and shipped to Mozart Therapeutics for processing to PBMCs and cryopreservation. Human intestinal tissue was collected from consented donors under contract with Benaroya Research Institute (IRB#10090).

### 2.14 Sequence alignments and percent sequence identity

Multiple sequence alignments were performed using EMBL-EBI Clustal Omega (20). Percent sequence identities were calculated using the Percent Identity Matrix tool in UniProt. Extracellular domains (ECDs) were determined from annotated UniProt entries for each protein. If UniProt entries were not available, ECDs were predicted based on homology to annotated KIR, Ly49, or CD8a UniProt entries.

### 2.15 Acute GvHD model

Human NSG mice (NOD.Cg-*Prkdc^scid^IL2rg^tm1Wjl^/SzJ*, obtained from Jackson Laboratories, Bar Harbor, ME, USA) were randomized into groups by weight and engrafted with 1 × 10^7^ healthy human donor PBMCs (Bloodworks Northwest) at 4 hours post-irradiation at the doses specified. One day post-engraftment, mice were treated with saline or MTX-101 intravenously and every 7 days for the duration as specified in study designs. Mice in the IL-2 control group were injected every other day intravenously with 25,000 IU IL-2 (R&D Systems) from day 0 to day 10 (21). Abatacept (supplied by Jackson Laboratories) control mice received 5 mg/kg intraperitoneally every 48 hours starting on study day 0 to study day 26 for a total of 14 doses. Whole blood was collected at the timepoints shown, stained for surface and intracellular markers following standard protocols (22), and evaluated using flow cytometry. At clinical and study terminal endpoints, spleen samples were also collected for flow cytometry.

### 2.16 Exploratory tolerability study and pharmacokinetic assessment

Female BALB/cJ mice (Strain number: 000651) aged 7-weeks and NOD.Cg-Prkdc^scid^ Il2rg^tm1Wjl^ Tg(IL15) /SzJ (CD34+ NSG-Tg(Hu-IL15), cat# 030890) mice at 12 weeks post engraftment were purchased from the Jackson Laboratory.

Forty-eight NSG-Tg(Hu-IL-15) mice at 4 weeks of age were engrafted with CD34+ cells from two donors (24 mice per donor; Donor 0818 and 0894). Twelve weeks post-engraftment, CD34+ NSG-Tg(Hu-IL-15) was screened for human CD45 (hCD45) cells, CD3+ T cells, and CD56+ NK cells in the peripheral blood. Only mice with >25% hCD45 cells, >3% CD3+ T cells, and >2% CD56+ NK cells were enrolled in the study. Predose bleed for CD34+ NSG-Tg(Hu-IL-15) mice was performed 15 weeks after engraftment. On the day of dosing, CD34+ NSG-Tg(Hu-IL-15) animals were 16 weeks post-engraftment. The two donor cohorts of female CD34+ NSG-Tg(Hu-IL-15) mice were administered via tail vein one dose of vehicle, MTX-101, single-arm anti-KIR, or single-arm anti-CD8 at 5 mg/kg or OKT3 (BioXCell, Lebanon, NH, USA, cat# BE0001-2) at 0.5 mg/kg (n = 3 per dosing group and donor). All animals were retro-orbitally bled at pre-dose (-336-hrs) and post-dose at 2, 24, 72-hrs. At the terminal timepoint of 168 hours, blood via cardiac puncture was collected from each animal under anesthesia. Samples were evaluated for pharmacokinetic (PK), cytokine analysis, and immunophenotyping.

In a separate study, two additional cohorts of CD34+NSG-Tg(Hu-IL-15) mice, as screened above, were used to collect miscrosample matrix samples over time at 0.5 hours, 2 hours, 8 hours, 24 hours, 96 hours, 168 hours, 252 hours, 336 hours, 420 hours, 504 hours, 588 hours, and 672 hours post-single dose of MTX-101 at 1 or 10 mg/kg. For microsamples, 10 µL of whole blood was diluted in assay buffer, cells were removed, and the supernatant was frozen at −80°C.

### 2.17 MSD cytokine analysis

Serum cytokine analysis was carried out using a human 6-spot U-PLEX MSD assay for IFN-γ, IL-2, IL-6, IL-10, MCP-1, and TNF-α according to the manufacturer’s instructions. The plate was read on the Meso QuickPlex SQ 120MM and analyzed using MSD Discovery Workbench (Meso Scale Diagnostics). Due to limitations in serum volume, each timepoint was conducted as single measurements.

### 2.18 PK analysis

Concentrations were determined using ELISA plates coated with soluble KIRs (sKIR, hKIR2DL1 ECD) to capture MTX-101 in the mouse matrix. An horseradish peroxidase (HRP)-labeled goat anti-human IgG antibody was then used to detect the captured MTX-101. Tetramethylbenzidine (TMB; VWR, Visalia, CA, USA) and TMB STOP solutions (VWR) were used to produce the colorimetric signal that was proportional to the amount of captured MTX-101. The plate was read at 450 nm with a reference wavelength of 650 nm using a spectrophotometer, and a standard curve was used to determine concentration with a five-parameter logistic (5-PL) regression model with 1/Y^2^ weighting. For PK analysis using CD34+ NSG-Tg(Hu-IL-15) mice, individual microsample collections from all animals per timepoint were analyzed for MTX-101 concentration. Duplicates were conducted for each timepoint within the sample sets. The MTX-101 concentration versus time data from the full PK study using CD34+ NSG-Tg(Hu-IL-15) study was imported into Phoenix WinNonlin v8.3.4.295 (Certara, Princeton, NJ, USA) for analysis to determine elimination PK parameters.

All *in vivo* studies were conducted under animal study protocols reviewed by the Institutional Animal Care and Use Committee (IACUC) of the testing facilities including Labcorp Corporation (Greenfield, IN, ACUA 21-203-B), and the Jackson Laboratory Research (Sacramento, CA, IACUC 20110 and 18003). For all animal studies, euthanasia was by terminal exsanguination and CO2 asphyxiation.

### 2.19 Statistics

Multiple sample comparisons were made using ANOVA, and two-way comparisons were performed using Student’s *t*-test in GraphPad Prism 10. For Incucyte experiments, comparisons of the area under the curve were made using ANOVA testing for the CD8 Treg dose group compared to the control group (no CD8 Tregs). For studies, bars represent mean expression across independent donors or experiments as specified in figure legends, and error bars denote standard deviation. In figures, *p < 0.5, **p < 0.01, ***p < 0.005, ****p < 0.001, and ns = not significant.

## 3 Results

**Figure 1:**
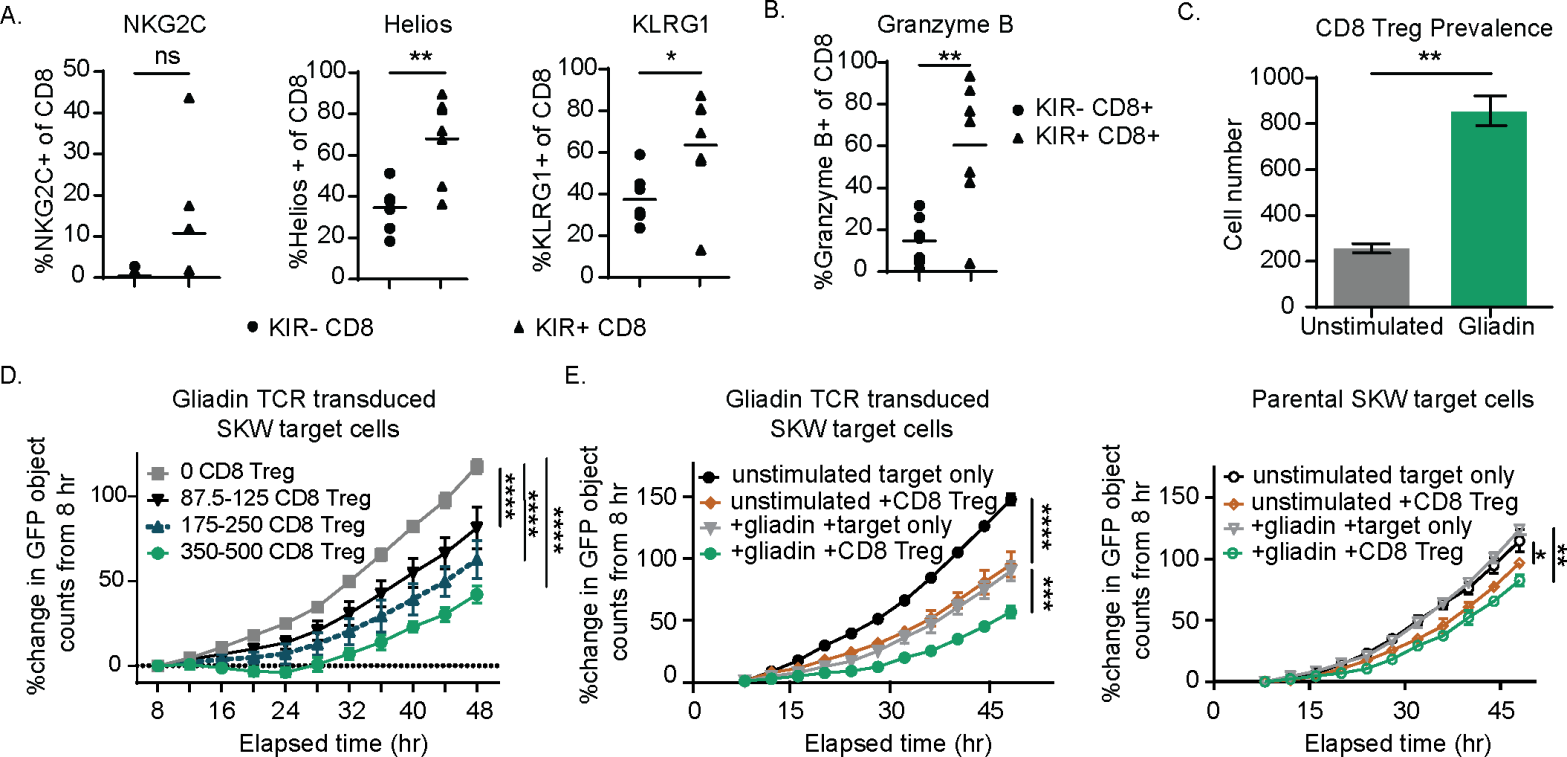
KIR CD8 regulatory T cells (Tregs) express the surface markers NKG2C, Helios, and KLRG1 and, when enriched from celiac donor peripheral blood mononuclear cells (PBMCs), specifically eliminate a gliadin T-cell receptor (TCR)-transduced SKW line in a dose-dependent manner. **(A, B)** Healthy donor PBMCs were plated overnight and then stained for KIRs, NKG2C, Helios, and KLRG1 **(A)** and the cytolytic marker, Granzyme B **(B)**. p-Values were determined with a paired *t*-test. **(B)** The percentage positive of each of these markers is shown for the KIR+ CD8 Treg and KIR− CD8 non-CD8 Treg populations from seven healthy donors. **(C)** KIR+ CD8 Tregs were isolated from celiac PBMCs following expansion with IL-7, IL-15, and gliadin peptides. Sorted CD8 Tregs were then cultured with gliadin-expanded CD4 T cells and autologous antigen-presenting cells (APCs) with or without (unstimulated) additional gliadin peptide stimulation for 3 days and evaluated for CD8 Treg number. Results are representative of two independent experiments across three different celiac donors. p-Values were determined with an unpaired *t*-test. **(D)** CD8 Tregs were enriched from celiac donor PBMCs following expansion in IL-7 and IL-15 for 7 days and sorting for CD5+NKp46-CD4-CCR7-CD28- T cells. CD8 Tregs were quantified, and increasing ranges of CD8 Tregs were cultured with 20,000 GFP+ gliadin-responsive TCR-transduced SKW target cells. Percent change in GFP+ objects (as determined by Incucyte software) from baseline (t = 8 hours) is shown for target cells in the absence of CD8 Tregs and with increasing CD8 Treg number. Results are representative of three independent experiments. **(E)** CD8 Treg-enriched cells were cultured with either green fluorescent protein (GFP)-expressing parental SKW or gliadin TCR-transduced SKW target cells for 48 hours and the percent change in GFP signal relative to baseline (t = 8 hours, n = 2 independent experiments). **(A–E)** ns: p > 0.05, *p < 0.05, **p < 0.01, ***p < 0.001, ****p < 0.0001. **(D, E)** p-Values were determined through a one-way ANOVA of calculated areas under the curves followed by Šídák’s multiple-comparisons test.

In individuals with autoimmune disease, CD8 Treg do not productively engage and eliminate autoreactive CD4 T cells. CD8 Treg dysfunction may be partially due to the expression of inhibitory KIRs, functioning as an auto-immune checkpoint. We hypothesized that blocking inhibitory KIR signaling may reverse dysfunction. Phenotypic analysis revealed that CD8 Tregs were enriched for expression of KLRG-1 and the transcription factor Helios relative to KIR-negative CD8 T cells (**Supplementary Figure 1**) and trends of higher expression of NKG2C in three out of seven donors (**Figure 1A**) (23). A higher percentage of CD8 Tregs were also positive for the cytolytic protein Granzyme B compared to KIR-negative non-Treg CD8 T cells in the absence of stimulation (**Figure 1B**), consistent with a mechanism of action previously described for CD8 Tregs (1). Gliadin peptide (**Supplementary Table 1**) restimulation of CD4 T cells derived from human donors with celiac disease induced a corresponding expansion of purified celiac donor CD8 Tregs when cultured with CD4 T cells (**Figure 1C**), consistent with data illustrating the expansion of CD8 Tregs in response to high-dose deamidated gluten (1). The use of immunodominant gliadin epitopes supports that CD8 Tregs can respond to oligoclonal pathogenic CD4 T-cell responses in the absence of inflammatory responses derived from other cell types (24).

To confirm that CD8 Tregs (**Supplementary Figure 2**) eliminate pathogenic CD4 T cells by direct killing as described (1), we used a green fluorescent protein (GFP)-labeled CD4+ SKW target cell line engineered to express an alpha1a-gliadin peptide specific TCR (**Supplementary Table 1**, 25). CD8 Tregs suppressed gliadin-specific TCR-transduced SKW cell expansion in a dose-dependent manner following gliadin stimulation (**Figure 1D**). CD8 Treg killing was specific to activated gliadin TCR-transduced SKW cells, as parental cells lacking the gliadin-specific TCR (parental SKW targets) were not eliminated as effectively in the presence of CD8 Tregs, even when activated (**Figure 1E**). Confirming the phenotypic signature of celiac patient-derived CD8 Tregs, we found that cultures depleted of CD8 Tregs did not eliminate gliadin-specific TCR-transduced SKW cells (**Supplementary Figures 3A–C**). Together, these results illustrate that CD8 Tregs derived from donors with celiac disease can directly and selectively kill gliadin-specific TCR-transduced CD4 T cells in a dose-dependent fashion.

### 3.1 MTX-101 binds to KIRs and CD8

**Figure 2:**
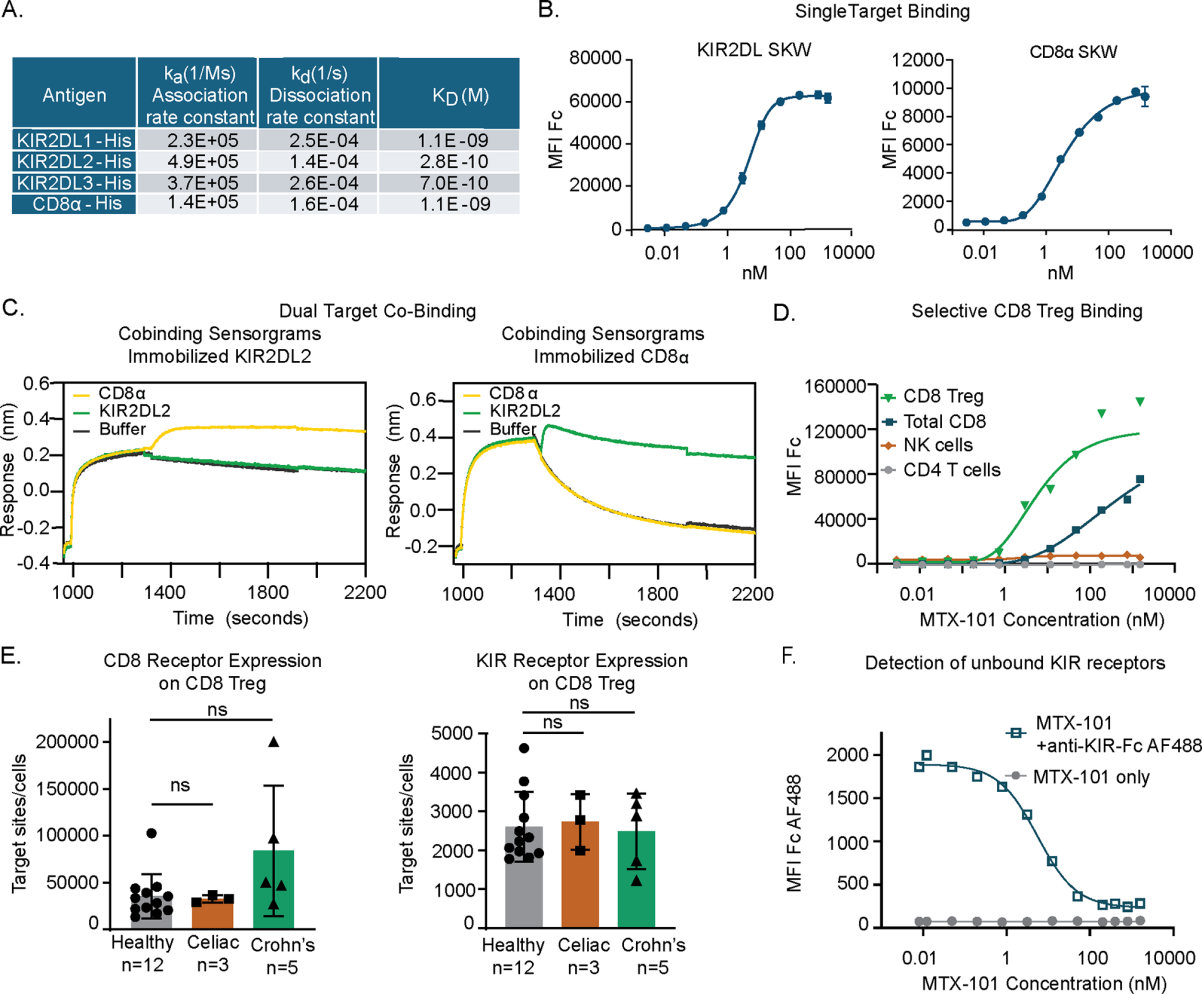
MTX-101 specifically binds to target surface receptors on CD8 regulatory T cells (Tregs). **(A)** Table shows the binding affinities of MTX-101 for human KIR2DL1/2/3 and CD8α based on association and dissociation rate constants measured for each binding interaction, and K_D_ binding affinity calculated from the measured rate constants. **(B)** Dose–response curves of MTX-101 binding to stably expressed KIR2DL1 and CD8α SKW cell lines. EC_50_ values were calculated for each cell line using Prism GraphPad software with non-linear regression [agonist vs. response curve (three parameters)]. Binding curves are representative of binding to KIR2DL1 expressing (n = 14 independent experiments) and CD8a expressing (n = 10) cell lines. **(C)** Simultaneous co-binding of antigens to MTX-101 as determined by Bio-Layer Interferometry. MTX-101 was captured with either KIR2DL or CD8α, and then additional antigen binding was detected with CD8α or KIR2DL, respectively. **(D)** Dose-dependent MTX-101 binding to immune cell populations within total human peripheral blood mononuclear cells (PBMCs) is shown as detected with an anti-human Fc secondary antibody. Data are representative of seven independent experiments with different donors. **(E)** Quantification of CD8 and KIR binding sites per CD8 Treg target cell as determined by receptor mean fluorescence intensity (MFI) and quantitative counting beads is shown in the figure. p-Values were determined through a one-way ANOVA of calculated areas under the curves followed by Šídák’s multiple-comparisons test. ns: p > 0.05. **(F)** Detection of unbound KIR sites with a saturating dose of fluorescently labeled single-arm KIR-Fc antibody on CD8 Tregs in total PBMCs following incubation with increasing doses of MTX-101. IC50 values were calculated for KIR-Fc using GraphPad Prism software and non-linear fit log (inhibitor) vs. response with variable slope and four parameters. The graph is representative of binding data across three independent healthy donors.

MTX-101 is a bispecific antibody containing a Fab that targets and blocks inhibitory KIRs (16) and a non-signaling, non-blocking scFv binding domain that targets CD8α (17), herein referred to as CD8). The binding, affinity, and kinetic rate constants of MTX-101 were evaluated. Single-target binding affinities to human CD8 or the three targeted isoforms of KIR2DL (KIR2DL1, KIR2DL2, and KIR2DL3) were found to be similar (**Figure 2A**, **Supplementary Figure 4A**). Association and dissociation rate constants were measured for each binding interaction, and K_D_ binding affinity was calculated from the measured rate constants (**Figure 2A**). On-cell target binding to inhibitory KIRs and CD8 was tested using SKW cell lines stably transduced to express KIR2DL (EC_50_ = 4.8 nM) or CD8 (EC_50_ = 4.1 nM) (**Figure 2B**). Binding to inhibitory KIRs was specific to the intended KIR family members, as binding to unmodified or KIR3DL1-expressing HEK 293 cells was not detected (**Supplementary Figure 4B**). Restricted KIR binding was confirmed using the Retrogenix cell microarray platform, in which HEK cells are transfected to express individual surface targets derived from a library of plasmids encoding over 6,500 human cell surface-expressed proteins as described (26), followed by binding of MTX-101 or relevant single-arm controls and flow cytometry analysis. This analysis revealed highly selective binding for the intended targets of KIRs and CD8 (**Supplementary Figure 4C**).

Co-binding of MTX-101 to CD8 and inhibitory KIRs was evaluated to ensure that the order of binding did not impact targeting. KIR2DL2 was utilized as the representative inhibitory KIR. In the first orientation, MTX-101 was captured onto immobilized KIR2DL2, followed by CD8 antigen binding (**Figure 2C**, left). For testing in the reverse orientation, MTX-101 was captured onto immobilized CD8, followed by KIR2DL2 antigen binding (**Figure 2C**, right). These results demonstrated that MTX-101 can simultaneously bind both inhibitory KIRs and CD8 antigens. Co-binding of MTX-101 to inhibitory KIRs and CD8 was also observed in PBMC binding assays where MTX-101 preferentially binds dual target-expressing CD8 Tregs relative to single target-expressing NK or non-Treg CD8 T cells (**Figure 2D**). As anticipated, no binding to CD4 T cells was observed at any concentration tested (**Figure 2D**).

MTX-101 binding measured by gMFI on CD8 Tregs is driven by the number of CD8 receptors expressed on the cell surface (**Figure 2E**) due to the higher number of CD8 receptors on the cell surface relative to KIRs on cells both derived from healthy and disease donors (i.e., celiac disease and Crohn’s disease) (**Figure 2E**). Given that the proposed mechanism of action of MTX-101 is dependent on blocking inhibitory KIRs, we determined the saturating concentration of MTX-101 for KIRs on CD8 Tregs. Using a labeled single-arm KIR-Fc molecule to probe for unbound KIRs following binding of increasing concentrations of MTX-101, we determined that saturation of MTX-101 to KIR binding occurred at a concentration greater than 100 nM, with an IC50 of 5.5 nM (**Figure 2F**).

### 3.2 *In vitro* functional assessments of MTX-101

**Figure 3:**
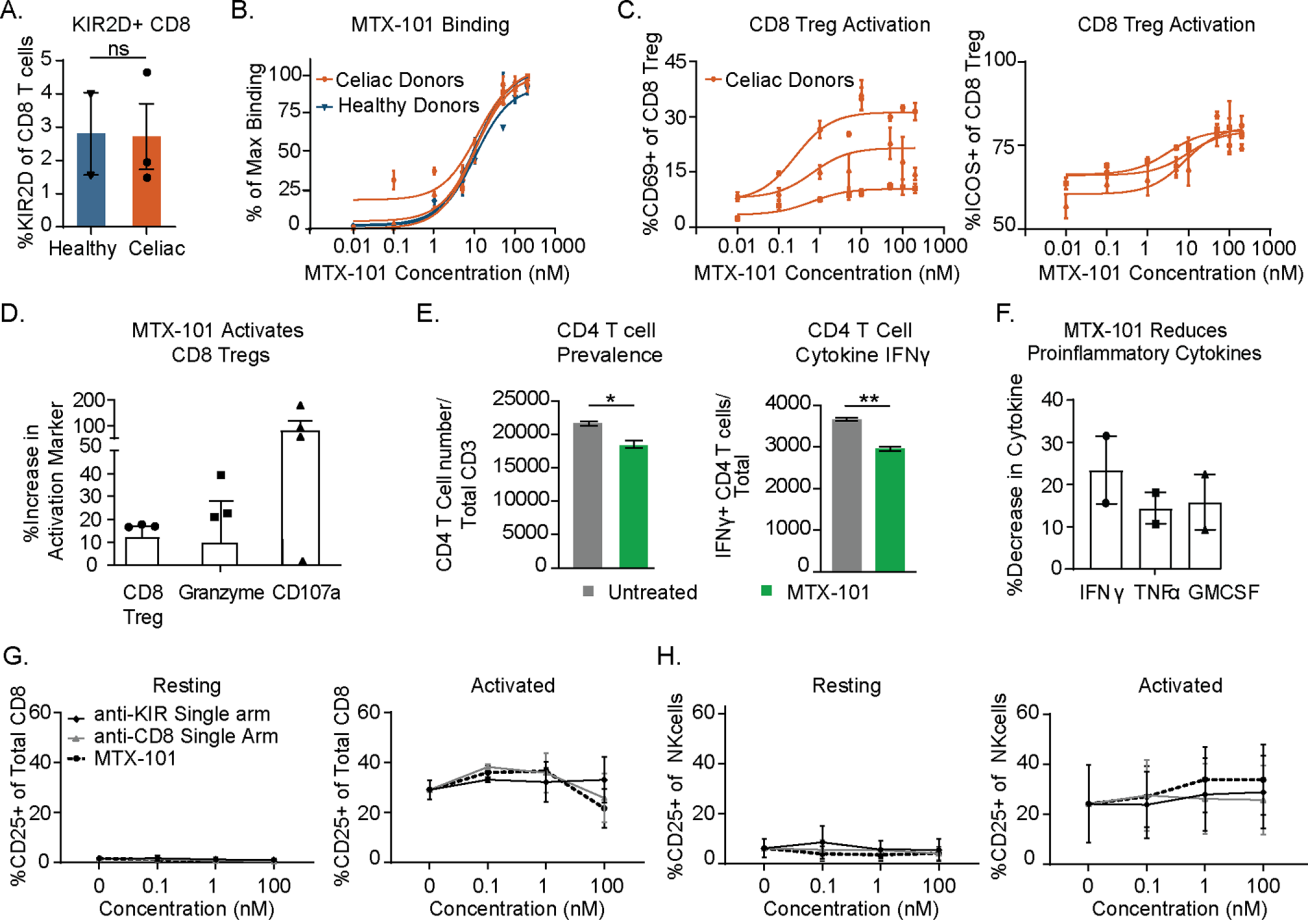
MTX-101 activates CD8 regulatory T cells (Tregs) in celiac and healthy peripheral blood mononuclear cells (PBMCs) and induces lysis of gliadin-specific target cells in celiac T-cell co-culture assay. **(A)** Percentage of CD8 Tregs detected in healthy (n = 2) and celiac (n = 3) PBMCs. **(B)** MTX-101 dose-dependent CD8 Tregs binding across the same donors as in panel **(A)** in total PBMCs. p-Values were determined with an unpaired *t*-test. ns: p > 0.05, *p < 0.05, **p < 0.01. **(C)** MTX-101 dose-dependent activation of CD8 Tregs in celiac PBMCs (n = 3 donors) following 48 hours of incubation. **(D, E)** Celiac KIR+ CD8 Tregs were expanded in IL-7+IL-15+ gliadin peptides. Sorted CD8 Tregs were then cultured with autologous gliadin peptide-expanded CD4 T cells and peptide-pulsed antigen-presenting cells (APCs) +/− 100 nM MTX-101 for 7 days. **(D)** Percentage of CD8 Treg and Granzyme B and CD107a expression in CD8 following MTX-101 treatment. **(E)** CD4 readouts in co-cultures including number of total CD4 T cells and IFN-γ-producing CD4 T cells following 72-hour incubation with MTX-101. **(F)** Meso Scale Discovery (MSD) quantification of secreted IFN-γ, TNF-α, and granulocyte-macrophage colony-stimulating factor (GM-CSF) in the supernatant of co-cultures stimulated with gliadin peptides in the presence of MTX-101 shown relative to untreated controls. **(D–F)** Results are representative of two independent experiments (n = 3 donors). p-Values were determined with an unpaired *t*-test. ns: p > 0.05, *p < 0.05, **p < 0.01. **(G, H)** Activation of CD8 T cells **(G)** or NK **(H)** cells as determined percentage CD25 positive is shown for two healthy donor PBMCs following 48-hour incubation in the shown concentrations of anti-KIR single-arm antibody, anti-CD8 single-arm antibody, or MTX-101 in either untreated (resting) or anti-CD3-treated (0.1 µg/mL, activated) PBMCs.

To determine the functional consequences of MTX-101 binding, we treated PBMCs derived from healthy donors and donors with celiac disease with MTX-101. In donors used for binding studies, the prevalence of CD8 Tregs in PBMCs (**Figure 3A**, **Supplementary Table 2**) and MTX-101 binding was comparable between healthy donors and celiac donors on a gluten-free diet (**Figure 3B**). MTX-101 treatment increased CD8 Treg activation in celiac donor-derived PBMCs, as measured by ICOS and CD69 expression (**Figure 3C**). To determine if CD8 Treg activation was MTX-101-dependent, we treated PBMCs with an anti-RSV control antibody in each instance, and we observed no CD8 Treg activation (**Supplementary Figure 5A**) (27). CD8 Treg surface receptor modulation (**Supplementary Figure 5B**) and killing of antigen-responsive CD4 T cells (**Supplementary Figure 5D**) was not associated with increased production of proinflammatory cytokines, TNF-α, IL-6, IFN-γ, and IL-17A following low-dose polyclonal T-cell stimulation (**Supplementary Figure 5E**).

Functional consequences of MTX-101 binding were tested using PBMCs derived from donors with celiac disease that were restimulated with gliadin peptides as in **Figure 1C**. MTX-101 increased the prevalence of CD8 Tregs, as well as CD8 Treg Granzyme B content and degranulation (**Figure 3D**, **Supplementary Table 2**). MTX-101 treatment reduced the frequency of gliadin peptide-responsive CD4 T cells and their IFN-γ production, determined by intracellular cytokine staining (**Figure 3E**), and reduced production of proinflammatory cytokines, i.e., IFN-γ, TNF-α, and granulocyte-macrophage colony-stimulating factor (GM-CSF) (**Figure 3F**, **Supplementary Table 2**).

Given that select NK cells express KIRs and non-Treg CD8 T cells express CD8, we tested the effect of single arm binding and the functional impact of MTX-101 on CD8 and NK cells in PBMCs. Single arm binding did not increase CD8 T-cell (**Figure 3G**) or NK-cell (**Figure 3H**) activation relative to the untreated controls in either resting or activated PBMCs across a dose titration of MTX-101.

**Figure 4:**
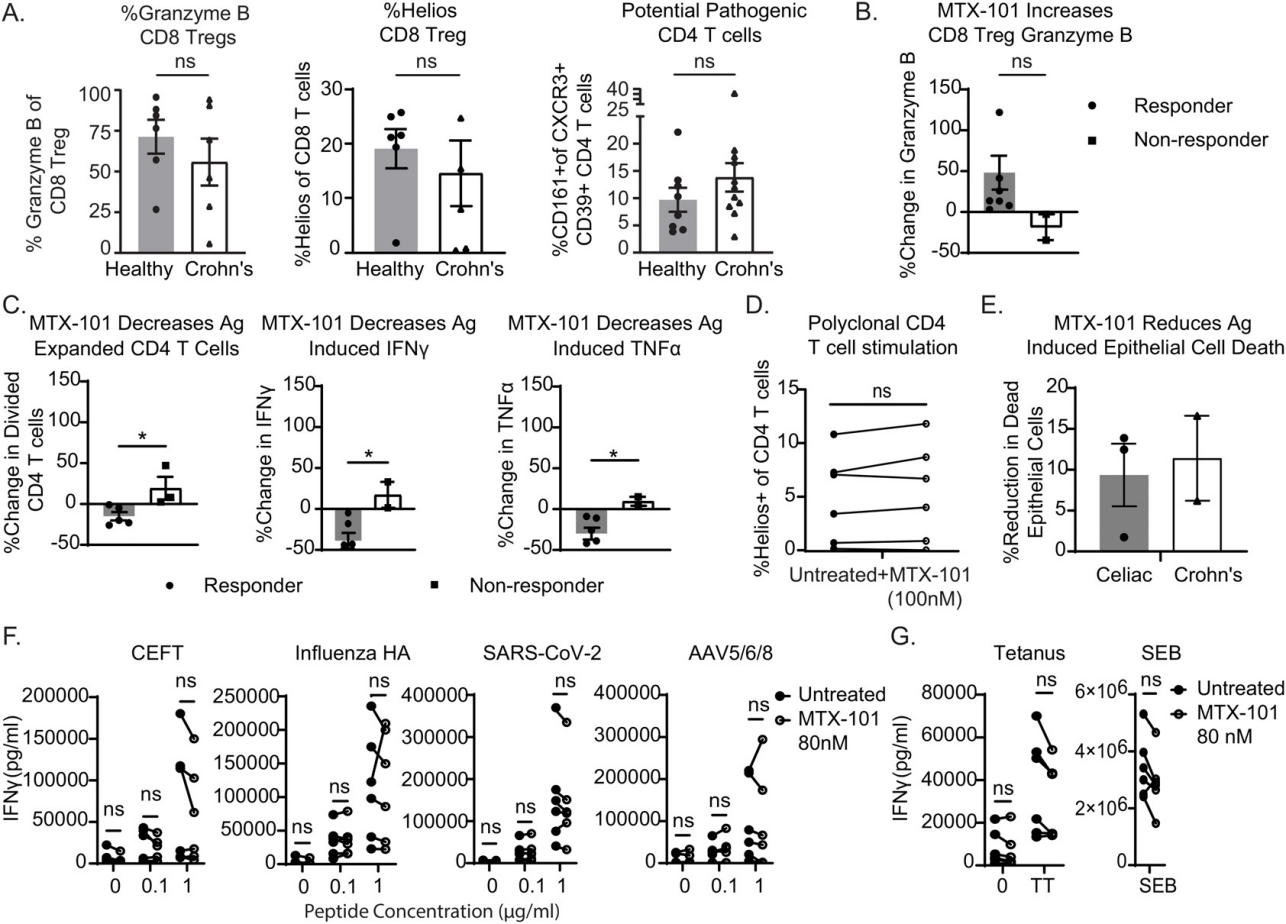
MTX-101 restores CD8 regulatory T-cell (Treg) functions and reduces antigen-induced proinflammatory responses in Crohn’s donor peripheral blood mononuclear cells (PBMCs) and organoids. **(A)** Healthy or Crohn’s donors (n = 6 each) CD8 Tregs were stained for Granzyme B and Helios. CD4 T cells were stained for co-expression of CD161+CXCR3+CD39+ in PBMCs from eight healthy (n = 8) or Crohn’s donors (n = 11). **(B, C)** Crohn’s PBMCs were incubated for 7 days with a mixture of IL-7+IL-15, bacterial flagellin, and OmpC peptide in the presence or absence of MTX-101 at 100 nM. **(B)** The percentage increase in concentration of Granzyme B in the supernatant of antigen-responsive or non-responsive Crohn’s donors (t = 5 days). **(C)** Carboxyfluorescein succinimidyl ester (CFSE) dilution of antigen-responsive CD4 T cells (t = day 7) antigen-expanded CD4 T cells as determined by diluted CFSE is also shown on day 7 for both responders and non-responders relative to untreated controls. Secreted IFN-γ and TNF-α (t = day 7) in MTX-101-treated donors relative to untreated. **(D)** Helios expression on CD4 T cells in PBMCs from Crohn’s donors stimulated with anti-CD3 (1 μg/mL) overnight in the presence and absence of MTX-101 (100 nM). **(A–D)** p-Values were determined with an unpaired *t*-test. ns: p > 0.05, *p < 0.05. **(E)** Change in antigen-induced epithelial cell death in both celiac and Crohn’s donor-derived organoids +/− 100 nM MTX-101. Results represent celiac (n = 3) and Crohn’s donors (n = 2). **(F)** Healthy or celiac PBMCs (n = 3 each) were restimulated with CEFT, Influenza HA, SARS-CoV-2, or AVV5/6/8 peptide (0, 0.1, or 1 µg/mL), in the presence and absence of MTX-101. IFN-γ detected in PBMC culture supernatant on day 5. **(G)** IFN-γ concentrations in supernatants following 5-day bacterial stimuli of tetanus toxoid (5 µg/mL) or SEB (100 ng/mL). Each point represents the mean of two technical replicates. Values untreated and with MTX-101 were compared using unpaired *t*-tests and two-way ANOVA followed by Šídák’s multiple-comparisons test.

We extended the functional characterization of MTX-101 to PBMCs derived from donors with Crohn’s disease. At baseline, half of the Crohn’s disease-derived CD8 Tregs showed decreased Granzyme B and Helios when compared to the average of healthy donors (**Figure 4A**), and 5 out of 11 Crohn’s donors had increased CD4 T cells expressing the surface markers CXCR3, CD39, and CD161 (**Figure 4A**, right), which are co-expressed on pathogenic autoimmune CD4 T cells (28). We activated pathogenic CD4 T cells in Crohn’s patient PBMCs using a mixture of flagellin and OmpC peptide as antigens (18, 19, 29, 30). In those patient PBMCs that responded to the antigenic peptides, MTX-101 increased Granzyme B secretion (**Figure 4B**), which was associated with a selective reduction of pathogenic CD4 T-cell expansion and proinflammatory cytokines IFN-γ and TNF-α (**Figure 4C**, **Supplementary Table 3**). In contrast, polyclonal CD4 T-cell responses were not inhibited (**Figure 4D**).

Santos et al. described that epithelial cell death can be induced in organoids derived from uninflamed patient tissue biopsies using antigens that activate pathogenic autoimmune CD4 T cells (31, 32). To test the effects of MTX-101 on tissue-resident CD8 Tregs, we used organoids derived from colonic or duodenal tissue biopsies from patients with Crohn’s or celiac disease, respectively. Organoids were expanded from primary intestinal tissue (**Supplementary Figure 6A**) and contained the tissues’ intrinsic NK-, B-, and T-cell populations (**Supplementary Figure 6B**), including CD8 Tregs. The phenotypes of these immune cells were comparable to those of peripheral blood immune cells (**Supplementary Figure 6C**). Upon antigenic peptide stimulation in both celiac and Crohn’s patient-derived organoids, MTX-101 bound and activated CD8 Tregs (**Supplementary Figure 6D**) and reduced activated CD4 T cells, proinflammatory cytokines (**Supplementary Figure 6E**), and antigen-induced intestinal epithelial cell death (**Figure 4E**, **Supplementary Table 3**), supporting that MTX-101 can also impact tissue-resident CD8 Treg functions.

To extend the observations that polyclonal CD4 T-cell responses remain unaffected in the presence of MTX-101 (**Figure 4D**), we evaluated changes in PBMC cytokines in response to viral and bacterial pathogens in the presence of MTX-101. No significant change in PBMC cytokines was observed in response to a CEFT peptide cocktail [including immunodominant epitopes derived from cytomegalovirus (CMV), Epstein-Barr virus (EBV), influenza, and tetanus toxoid], influenza HA, SARS-CoV-2, and adenoviral vectors (**Figure 4F**) or to bacterial antigens derived from tetanus and *Staphylococcus* enterotoxin B (**Figure 4G**), supporting that MTX-101 may not affect CD4 T-cell responses directed toward a bacterial or viral challenge.

### 3.3 Cross-reactivity of MTX-101 binding to its targets in other species

MTX-101 species cross-reactivity was assessed using human, cyno, and mouse KIR/Ly49F and CD8 by Bio-Layer Interferometry. KIR species cross-reactivity data indicated that MTX-101 did not bind to cyno KIR or the Ly49F mouse ortholog of KIR (**Supplementary Figure 7A**). CD8 species cross-reactivity data indicated that MTX-101 did cross-react to cyno CD8 but not mouse CD8 (**Supplementary Figure 7B**). Multiple sequence alignments and corresponding percent sequence identities of the human, cyno, and mouse KIR/Ly49 (**Supplementary Figure 8**, **Supplementary Table 4**) or CD8 (**Supplementary Figure 9**, **Supplementary Table 5**) extracellular domains substantiated the lack of species cross-reactivity by confirming the low overall sequence homology between the KIR or CD8 orthologs.

### 3.4 MTX-101 activity in a mouse model of inflammation

**Figure 5:**
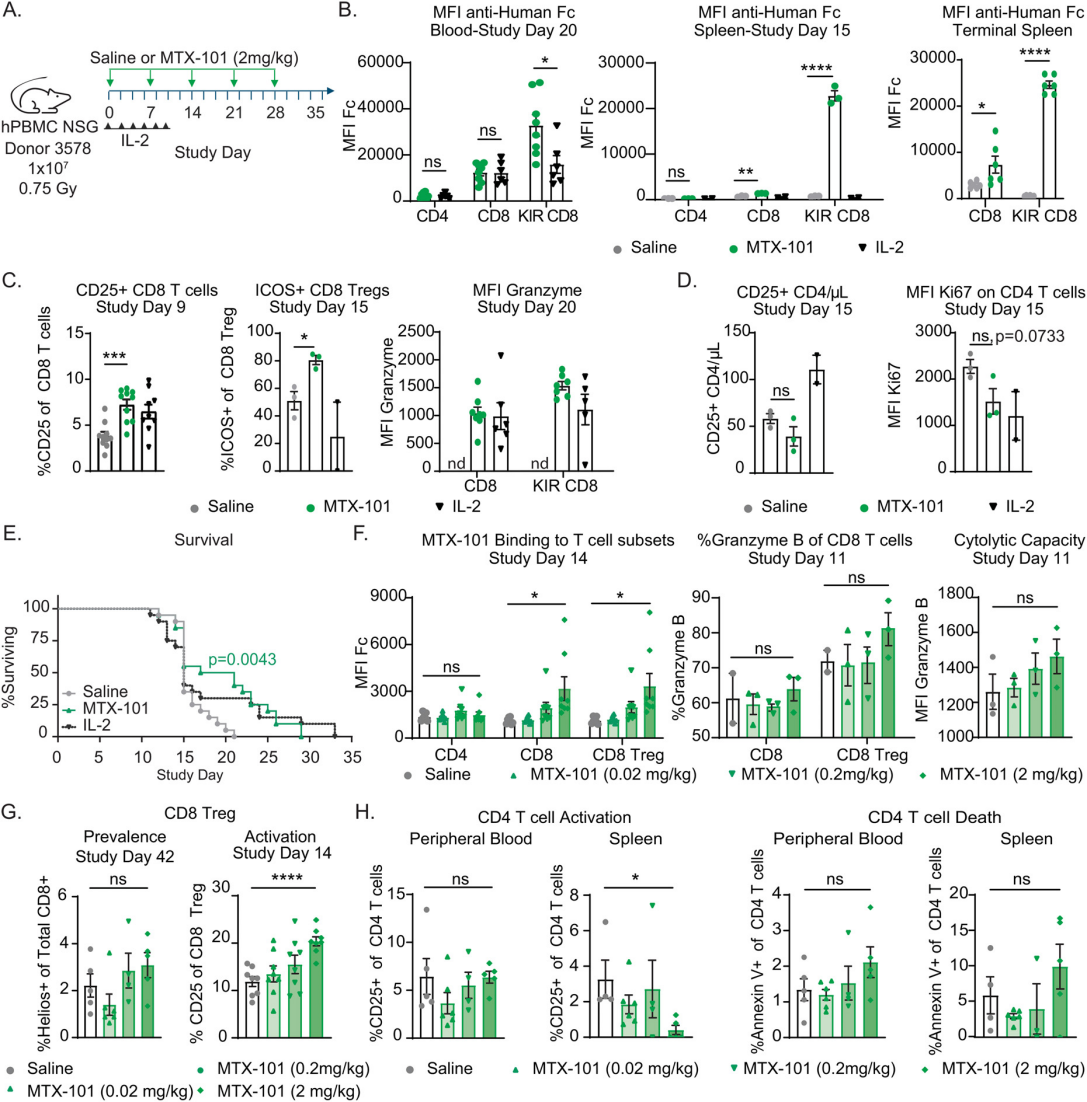
MTX-101 binding, efficacy, mechanism of action, and dose titration in acute graft-versus-host disease (GvHD) human PBMC-engrafted NSG mouse model. **(A)** Survival study experimental design. NSG mice were irradiated (0.75 Gy) and injected intravenously with 1 × 10^7^ human PBMC healthy donor 3578 (haplotype HLA-DQ2.5). MTX-101 (2 mg/kg) or saline was injected every 7 days until day 28. Low-dose IL-2 (25,000 IU) was injected every other day from study day (SD) 0 to 10, n = 20/cohort. **(B)** MTX-101 binding to CD4, CD8, and CD8 regulatory T-cell (Treg) populations as detected by mean fluorescence intensity (MFI) of anti-human Fc antibody in the blood on SD20, and spleens were collected on SD15 (MTX-101 and saline, n = 3 mice; IL-2, n = 2 mice) and at study termination (MTX-101 and saline, n = 6 mice). SD20 timepoint is representative of mice remaining in the MTX-101 (n = 8) and IL-2 treatments (n = 6). **(C)** CD25 and ICOS-positive CD8 non-Tregs and CD8 Tregs in peripheral blood as detected on SD9 and 15 (n = 10/cohort). CD8 Tregs and CD8 non-Treg Granzyme B MFI on SD20. nd: not determined. **(D)** Absolute counts of CD25+CD4 T cells/μL of blood on SD15 (MTX-101 and saline, n = 3 mice; IL-2, n = 2 mice). Ki67 MFI in CD4 T cells in peripheral blood on SD15. **(A–D)** p-Values were determined with an unpaired t-test. ns: p > 0.05, *p < 0.05, **p < 0.01, ***p < 0.001, ****p < 0.0001. **(E)** Survival curve in the acute GvHD model (saline, MTX-101, and low-dose IL-2, n = 18/cohort). Survival curves were compared using the log-rank (Mantel–Cox) test. p=0.0043. **(F–H)** Human NSG mice engrafted with 1 × 10^7^ human peripheral blood mononuclear cells (PBMCs) following irradiation (0.5 Gy) from donor LZ0007 and dosed with increasing concentrations of MTX-101 intravenously every 7 days on SD0–SD21. **(F)** Binding of MTX-101 to CD4, CD8, and CD8 Tregs (KLRG1+ CD8 T cells) in the blood on SD14. Granzyme B (MFI and percentage) on non-Treg CD8 and CD8 Treg populations in the blood and spleens from mice that were terminated on study day 11 (n = 3 mice). **(G)** CD25-positive CD8 Tregs and Helios-positive CD8 T cells in peripheral blood on SD14 (n = 8) and all surviving mice on SD42. **(H)** Percentage of CD25+ and Annexin V+ CD4 T cells on SD42 in peripheral blood and splenocytes. **(F–H)** p-Values were determined by an unpaired *t*-test. ns: p > 0.05, *p < 0.05, **p < 0.01, ***p < 0.001, ****p < 0.0001.

To evaluate MTX-101 effects in a highly inflammatory setting *in vivo*, we tested MTX-101 in an aggressive, systemic inflammatory humanized murine model of acute graft-versus-host disease (GvHD) that engrafts human T cells, including CD8 Tregs (**Figure 5A**) (33). In contrast to abatacept, which is approved for the treatment of rheumatoid arthritis and ablates lymphocyte engraftment (34, 35), MTX-101 treatment did not reduce human PBMC engraftment in this model (**Supplementary Figure 10A**), making the humanized GvHD model a viable option for evaluation of MTX-101 effects on inflammation *in vivo*

MTX-101 was selectively bound to CD8 Tregs in the peripheral blood and spleen, and binding was sustained between doses (**Figure 5B**). MTX-101 significantly increased CD8 Treg activation and Granzyme B content (**Figure 5C**, **Supplementary Figure 10B**), as well as trends in reduced proliferation and prevalence of activated CD4 T cells (**Figure 5D**). Weekly MTX-101 administration through day 28 significantly extended survival relative to saline-treated controls (**Figure 5E**). Cohort survival was consistent with a cohort treated with low-dose IL-2 (**Figure 5E**), a CD4 Treg-directed immune therapy used clinically to treat patients with steroid-refractory GvHD (36) or autoimmunity (37). In a separate study, trends in reduction in serum concentrations of the proinflammatory cytokine, IFN-γ (**Supplementary Figure 10C**), clinical disease scores, and pathology in disease-affected tissue were observed (**Supplementary Figure 10D**). Results supporting the MTX-101 mechanism of action (including binding to CD8 Tregs but not CD4 T cells, increase in CD8 Treg intracellular Granzyme B, and increase in CD8 Treg prevalence, activation, and cytolytic capacity) were confirmed to be dose-dependent (**Figures 5F, G**). These observations corresponded to a dose-dependent reduction of activated CD4 and an increase in CD4 T-cell death in both peripheral blood and spleens of MTX-101-treated mice that persisted until study termination (**Figure 5H**). Efficacy and mechanistic readouts were consistent across four repeat studies using three different donors (**Supplementary Figures 11A–H**), including confirmed MTX-101 binding to CD8 Tregs up to 7 days after the last 2 mg/kg dose (**Supplementary Figure 11H**). Collectively, these data support the anti-inflammatory effect and proposed mechanism of action for MTX-101 that was observed *in vitro*.

### 3.5 Assessment of binding and pharmacokinetics of MTX-101 in a humanized transgenic mouse model

Due to species-specific evolution and diversity of KIRs in non-human primates (38) and the lack of KIR expression in mice (39), we evaluated the binding and pharmacokinetics of MTX-101 *in vivo* using humanized NSG mice engrafted with CD34+ cells derived from human umbilical cord blood that are transgenic for human IL-15 cytokine (CD34+ NSG-Tg(Hu-IL-15) (40). These mice have physiologic serum levels of human IL-15, which supports the engraftment of a broad range of human immune cell subsets, including NK cells (**Supplementary Figure 12A**).

**Figure 6:**
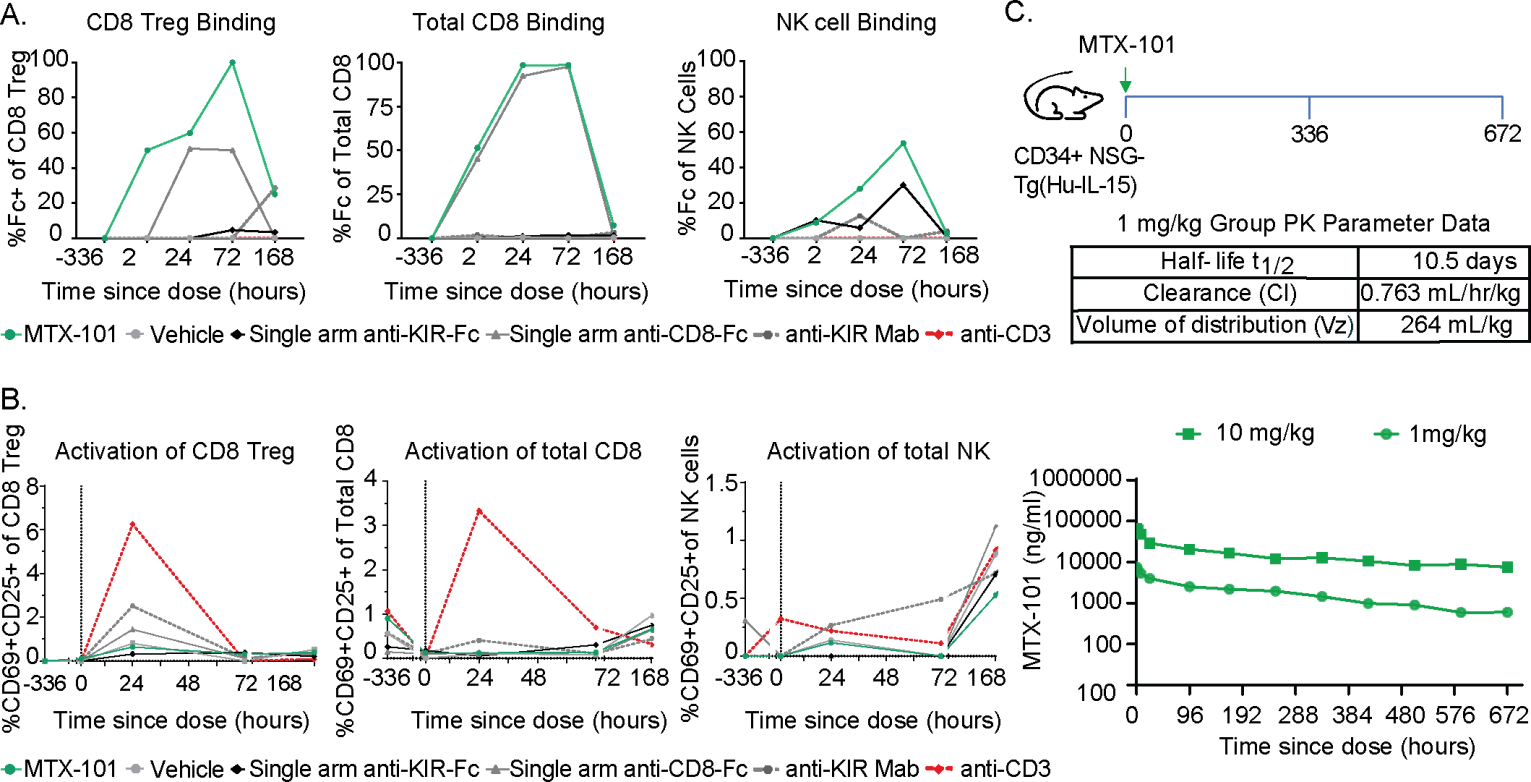
MTX-101 treatment does not activate CD8 or NK cells *in vitro* or in humanized CD34+ NSG-Tg(Hu-IL-15) mice despite antibody-like half-life in the serum. **(A)** Binding of MTX-101, single-arm anti-KIR, single-arm anti-CD8, or anti-KIR bivalent monoclonal antibody to CD8 regulatory T cells (Tregs) (left), non-Treg CD8 T cells (center), and NK cells (right) after a single dose of 5 mg/kg. Data are representative of two samples of pooled blood from three mice that received CD34 cells from two different donors at baseline, 3 hours, 24 hours, and 72 hours and all six mice at 168 hours. **(B)** Activation marker CD69+/CD25+ expression on CD8 Tregs (left), non-Treg CD8 T cells (center), and NK cells (right) after one 5 mg/kg dose of MTX-101, single-arm anti-KIR, anti-CD8 antibody, bivalent anti-KIR monoclonal antibody, or anti-CD3 (OKT3) control antibody at 0.5 mg/kg. **(C)** *In vivo* study experimental design. Humanized CD34+ NSG-Tg(Hu-IL-15) were injected with 1 and 10 mg/kg MTX-101, and blood was collected at specified timepoints in the diagram. Exposure of MTX-101 in CD34+ NSG-Tg(Hu-IL-15) was quantified from at start to 672 hours. Table shows the half-life of MTX-101, clearance rate, and volume distribution.

In concordance with the observation of MTX-101 binding *in vitro* and the humanized GvHD model, a single dose of 5 mg/kg MTX-101 had sustained binding to CD8 Tregs in peripheral blood relative to single-arm anti-KIR and anti-CD8 controls, with detectable binding at study termination 168 hours after dosing (**Figure 6A**). In contrast, MTX-101 binding to non-Treg CD8 T cells and NK cells had completely declined to baseline by the conclusion of the study (**Figure 6A**). MTX-101 binding to CD8 Tregs was greater than that detected on total CD8 T cells within terminal splenocyte samples (**Supplementary Figure 12B**), consistent with *in vitro* data (**Figure 2D**) and illustrating preferential binding of MTX-101 to immune cells expressing both targets. Despite binding to total CD8 T cells and NK cells, changes in activation (**Figure 6B**) or proinflammatory cytokines (**Supplementary Figure 12C**) were within baseline fluctuations and significantly less compared to controls in which either anti-CD3 or single-arm anti-KIR was administered.

In the presence of single and dual target expressing human immune cells, MTX-101 maintained a pharmacokinetic (PK) profile in CD34+ NSG-Tg(Hu-IL15) mice consistent with that observed in Balb/c mice (data not shown) that lack target expression. Overall, the results generally followed a standard dose response with appropriate proportional results across the two doses (1 mg/kg and 10 mg/kg) (**Figure 6C**). After a single intravenous dose (1 mg/kg) in CD34+ NSG-Tg(Hu-IL-15) mice, MTX-101 half-life was determined to be 10.5 days with a clearance rate of 0.763 mL/hr/kg and volume distribution of 264 mL/kg (**Figure 6C**), which is within the range of half-life for antibodies (41).

## 4 Discussion

The presence of CD8 Treg populations in healthy donors implies a role for CD8 Treg function in controlling the expansion of potentially harmful CD4 T cells. In mice, depletion of CD8 Tregs expressing the KIR functional ortholog, Ly49, has increased autoimmune disease pathology yet retains sufficient antiviral responses (1), an observation potentially linking CD8 Tregs to autoimmunity. Consistent with our data and those of others (8, 42, 43) illustrating cytolytic functions, previous work has demonstrated that CD8 Treg functions are perforin-dependent (8). We hypothesized that in autoimmune disease, serial responses to autoimmune disease triggers may further impair CD8 Treg function. However, interestingly, dysfunction of CD8 Tregs appears different than functional exhaustion, as demonstrated by differences in expression markers (23, 43), the failure of CD8 Tregs to control pathogenic autoimmune CD4 T cells despite retaining their cytolytic capacity in autoimmune patients, and their ability to regain cytolytic functions as we demonstrate and has been described by others (1, 44). The role of CD8 Treg function in contrast to exhaustion in autoimmunity is also supported by observations that adoptive cell transfer of subsets of CD8 T cells is associated with the reduction of inflammation and amelioration of autoimmune disease severity in experimental models (8). An increased prevalence of CD8 Treg has been described in patients with autoimmune disease that recover functions when activated ex vivo by others (1) and as we present here. Lastly, increases in functional CD8 Tregs are associated with improved outcomes in patients who respond to immune system-targeted therapies such as teplizumab (9), fingolimod (11), and low-dose IL-2 (37).

KIRs are known to maintain and regulate NK-cell self-tolerance (45). The association of genomic expression of KIRs with various autoimmune diseases (46, 47), coupled with the expression of inhibitory KIRs by CD8 Tregs, suggests that KIRs may also regulate CD8 Treg functions. KIRs compete with TCRs for binding to the MHC class I/peptide complex, and they are autoimmune checkpoints initiating an inhibitory signaling cascade that counteracts activating stimuli (14). Based on collective observations by us and others in animal models and patients with autoimmune diseases, we hypothesized that CD8 Treg functions can be restored by reducing the inhibitory signals delivered by KIRs, leveraging the selective reduction of pathogenic CD4 T cells, and mitigating the risk of broad immune suppression or off-target toxicities.

To that effect, we describe the preclinical characterization of MTX-101, a novel CD8 Treg modulator that is a bispecific antibody directed toward CD8 and inhibitory KIRs, both co-expressed on CD8 Tregs. Our data extend on previous work illustrating the dependence on CD8 Tregs to control EAE in a syngeneic model and the elimination of pathogenic gluten-responsive CD4 T cells in PBMCs derived from donors with celiac disease (1, 8). Advancing these findings by others, we link a phenotypic signature (**Figure 1A**) to functional elimination using Incucyte live cell imaging (**Figure 1D**, **Supplementary Figure 3**), confirming direct pathogenic CD4 T-cell lysis without an effect on polyclonally stimulated CD4 T cells (**Figure 1E**). The observation that polyclonal CD4 T-cell responses were unaffected in the presence of MTX-101 (**Figure 4D**) is consistent with a previous report demonstrating that mice deficient in CD8 Tregs did not have impaired antiviral responses (1).

Here, we show that the MTX-101-mediated inhibition of inhibitory KIR on CD8 Treg enhances their activation, Granzyme B content, and ability to eliminate activated pathogenic CD4 T cells derived from individuals with autoimmune disorders (**Figures 3**, **4B, C**). This enhanced elimination of autoreactive CD4 T cells results in reduced antigen-induced CD4 T-cell activation and epithelial cell death in tissues affected by autoimmune diseases (**Figure 4E**) and in a persistently inflammatory mouse model (**Figure 5**). The mechanism of action of MTX-101 aligns with the known biology of the CD8 Treg network (1), suggesting that MTX-101 effectively restores the primary function of CD8 Tregs to eliminate potentially pathogenic cells.

Our data also illustrate that MTX-101 selectively targets a relatively rare population of CD8 T cells expressing inhibitory KIRs, despite the presence of an abundance of single target-expressing non-Treg CD8 T and NK cells. By incorporating a non-functional CD8 targeting arm, MTX-101 demonstrates a preference for binding to and activating CD8 Tregs over non-Treg CD8 T and NK cells (**Figure 2D**), distinguishing it from the bivalent monoclonal antibody lirilumab, which elicits NK-cell activation through preferential NK-cell binding (48). MTX-101 testing in humanized mouse models exhibits a PK profile that is similar to what has been observed for monoclonal antibodies (**Figure 6C**), with no adverse activation of immune cells or induction of proinflammatory serum cytokines (**Figure 6B**, **Supplementary Figure 12C**). The safety of inhibitory KIR blockade observed with MTX-101 is consistent with the clinical testing of an inhibitory bivalent KIR monoclonal antibody tested in an oncology setting, in which there were no dose-limiting toxicities observed following treatment (48).

Preclinical studies have limitations, and the effect of MTX-101 on the CD8 Treg network continues to be evaluated and characterized in a Phase 1 clinical study (https://clinicaltrials.gov/study/NCT06324604). The CD8 Treg network represents a novel therapeutic approach. Although depletion of the CD8 Treg population appears specific for pre-disposing animals to autoimmune diseases (1), long-term consequences and conditional challenges in syngeneic, sterile animals have not been evaluated in such systems to our knowledge. The safety of targeting CD8 Tregs is consistent with the observed association of functions, prevalence, and the potential contribution of CD8 T-cell subsets to successful immune-targeted therapies in autoimmune patients. However, the possibility that such therapies have implications on immune responses that have yet to be observed cannot be ruled out.

Characterizing preclinically a bispecific molecule that targets a highly species-specific immune modulatory receptor has significant limitations. To address this we deploy primary human-derived materials from both healthy donors and autoimmune disease patients, as well as humanized animal models in our studies, with the anticipated consequence of diverse responsiveness to both antigen stimulation and MTX-101 treatment. Despite efforts to select common features of patient samples, such as current or past treatment regimens, disease state, and haplotype, human samples are heterogeneous. In contrast to homogeneous settings provided by syngeneic models, the variability of factors in human samples such as genetics, age, time since diagnosis, and other unknown characteristics impacted cohort size and limited statistical power and achievement of statistical significance.

Finally, although the receptors targeted by MTX-101, KIRs and CD8, are widely expressed across individuals and indications, variability in genomic copy number and regulation may depend on undefined factors, resulting in variable surface expression (**Figure 2E**) that may impact the effects of MTX-101 on CD8 Treg activation. Preclinical studies have limitations, and the effect of MTX-101 on the CD8 Treg network continues to be evaluated and characterized in a Phase 1 clinical study (https://clinicaltrials.gov/study/NCT06324604). The pre-clinical characterization will complement data derived from testing MTX-101 in patients and will improve understanding of the mechanisms governing the efficacy of MTX-101 in a fully human system.

## Data Availability

The original contributions presented in the study are included in the article/**Supplementary Material**. Further inquiries can be directed to the corresponding author.

## Supplementary material

Supplementary Figure 1 Gating strategy for the identification of KIR^+^ CD8^+^ T cells. **(A)** Single lymphocytes were selected using SSC-A/FSC-A and SSC-A/SSC-H parameters respectively. Dead cells were excluded using LIVE/DEAD Fixable Aqua. CD8^+^ T cells were selected based on co-expression of CD8α and CD5. KIR^+^CD8^+^ and KIR^-^CD8^+^ T cells were identified phenotypically by co-staining for KIR antibodies.

Supplementary Figure 2 Incucyte gating strategy and class I haplotype for CD8 Treg and gliadin TCR transduced and parental SKW target cells. **(A)** CD8 Treg enriched cells were sorted from celiac patient T cells following expansion in IL-7 and IL-15. Single cells were selected using FSC-H/FSC-A. Lymphocytes were selected using SSC-A/FSC-A. Dead cells were excluded using LIVE/DEAD Fixable Aqua. T cells were identified based on CD5 and NK cells were excluded based on NKp46. CD4 T cells were excluded. CCR7 and CD28 positive CD4- T cells were also eliminated to enrich for KIR+ CD8 T cells. The sorted population CD5+/NKp46-/CD4-/CCR7-/CD28- was then stained to determine %KIR+CD8. This percentage was then used to calculate total number of KIR+ CD8 T cells added to each incucyte well and CD8 Treg to target SKW ratios based on the initial number of SKW targets added to each well. **(B)** The MHC Class I haplotype of SKW target cells and celiac donor PBMCs used in the Incucyte assay.

Supplementary Figure 3 CD8 T cells enriched for Treg eliminate activated gliadin-responsive CD4 targets. **(A–C)** CD8 T cells were enriched from celiac donor PBMC following expansion in IL-7 and IL-15 for seven days and sorted on the surface markers KLRG1+CD244+CD28- (CD8 Treg enriched effectors) or KLRG1-CD244-CD28+ (non-CD8 Treg effectors). CD8 enriched effectors were combined with activated gliadin-responsive GFP+ SKW CD4 target cell line at a 2 to 1 ratio. Percent change in GFP+ objects from an 8 hour time point is shown for both CD8 Treg and non-CD8 Treg effectors over 48 hours cultured with targets. Activated targets only in the absence of addition of CD8 effectors are shown as a representative control. **(B, C)** Incucyte-generated images show the decrease in GFP+ gliadin-responsive SKW target cells over 48 hours for both CD8 Treg enriched **(B)** and Non-CD8 Treg effector cells **(C)**.

Supplementary Figure 4 MTX-101 binding is dose-dependent and specific to target proteins. **(A)** Dose-dependent binding curves for MTX-101 to KIR2DL1/2/3 and CD8α target antigens along with association and dissociation curves as detected by Bio-Layer Interferometry. **(B)** MTX-101 binding to KIR3DL1 transfected HEK 293 or untransfected cells. Dose-dependent binding of a KIR3DL1 targeted bispecific antibody is shown as a positive control on the KIR3DL1 transfected cell line. **(C)** Retrogenix live cell microarray technology study evaluated surface protein library transfected 293T cell binding of MTX-101 and single arm controls. Summary table of target binding is shown. Each KIR isoform tested and binding to MTX-101 or single arm controls (KIR Fab or CD8 scFv) is shown for both fixed and unfixed samples. Binding summary of CD8α is also shown for MTX-101 and controls. Of the 6,500 proteins tested there was also weak binding to Frizzled Class Receptor 5 (FZD5) as shown in the table. Strong and medium binding is coded as green in the table, weak binding is coded as blue, very (v.) weak binding is yellow, and no binding (neg) is white.

Supplementary Figure 5 CD8 Treg activation is not induced with control anti-RSV antibody and MTX-101 does not increase pro-inflammatory cytokine production in PBMC culture in the presence of anti-CD3 stimulation. **(A)** Percentage of CD69 positive CD8 Treg in healthy donor PBMC as detected by flow cytometry following incubation with no antibody, anti-RSV, or MTX-101 for 48 hours across two healthy donors. P values were determined by an unpaired t test. p=0.072, ns: p>0.05. **(B)** CD8 Treg prevalence, Granzyme B MFI, and degranulation quantified by CD107a expression following incubation of celiac donor CD8 Treg with CD4 target cells (1:5) and APCs in the presence or absence of 0.02 µg/ml anti-CD3 and MTX-101 (100nM) for three days. **(C)** IFNγ, TNFα, and GMCSF concentrations detected following the three day CD8 Treg and CD4 target co-culture as described in **(B)** in the presence of anti-CD3 with and without addition of MTX-101. **(B, C)** P values were determined by a paired t test. ns: p>0.05, *: p<0.05. **(D)** PBMC from two celiac donors were incubated with 0.8, 8 or, 80 nM of MTX-101 in the presence of low dose anti-CD3 (0.1 ug/mL) for 48 hours and endpoint %Annexin+ CD4s are shown. **(E)** Concentrations of the pro-inflammatory cytokines (TNFα, IL-6, IFNγ, and IL-17a) detected in the supernatant after incubation with low dose anti-CD3 in the presence or absence of MTX-101 for 48 hours across two different celiac donors.

Supplementary Figure 6 Crohn’s Organoids expand from primary intestinal tissue samples and contain relevant immune cells. **(A)** Representative image of Crohn’s intestinal tissue derived organoids. **(B)** Percentage of epithelial (EpCAM+), T, B, and NK cells present in both blood and primary intestinal tissue (colon) of 7 different Crohn’s donors at baseline. T cell graph shows the percentage of CD4 and CD8 T cells detected in the blood and colon tissue at baseline. **(C)** The KIR2DL and CD8 receptor quantification per CD8 Treg in the blood and colon as determined by receptor MFI and quantitation beads. Results are representative of 3 independent Crohn’s donors. **(B, C)** P values were determined by a unpaired t test. ns: p>0.05, *: p<0.05, ** p<0.01, *** p<0.001, ****p<0.0001. **(D)** MTX-101 binding as detected with an anti-human IgG Fc antibody is shown for an MTX-101 treated well relative to an organoid well that did not receive MTX-101 (Ag), left. Percentage increase in CD8 Treg activation (CD25) as determined relative to the untreated control at study endpoint. n=3 independent experiments. **(E)** The reduction in antigen induced CD4 expansion following treatment with MTX-101 is shown across celiac and Crohn’s organoid cultures n=4 independent experiments (left). Percentage reduction in IL-17a and GMCSF detected in organoid supernatant following MTX-101 treatment n=2 celiac donors.

Supplementary Figure 7 MTX-101 lacks cross-reactivity to cyno KIR, murine Ly49F, and murine CD8α and shows some cross-reactivity to cyno CD8α. **(A)** Binding of 400 nM MTX-101 to KIR target antigens along with association and dissociation curves as detected by Bio-Layer Interferometry. **(B)** Binding of 400 nM MTX-101 to CD8α target antigens along with association and dissociation curves as detected by Bio-Layer Interferometry. **(A, B)** Tables show the association and dissociation rate constants measured for each binding interaction, and K_D_ binding affinity calculated from the measured rate constants.

Supplementary Figure 8 Sequence alignment of the human and cyno KIR and mouse Ly49F extracellular domains generated by Clustal Omega. GenBank Accession numbers represent the sequence sources of either the commercially available hKIR or in-house generated cyKIR and mLy49F reagents used in the Octet cross-reactivity binding assay. A ‘-’ indicates a gap in the sequence alignment and a space indicates a non-conservative substitution.

Supplementary Figure 9 Sequence alignment of the human, cyno and mouse CD8α extracellular domains generated by Clustal Omega. GenBank Accession numbers represent the sequence sources of the commercially available CD8α reagents used in the Octet cross-reactivity binding assay. A ‘-’ indicates a gap in the sequence alignment, a ‘*’ indicates the residue at that position is identical in all three sequences, a ‘:’ represents a conservative substitution, a ‘.’ represents a semi-conservative substitution at that position and a space indicates a non-conservative substitution.

Supplementary Figure 10 Confirmation of human CD45 engraftment and dose-dependent decrease of Granzyme B in clinical endpoint mice. **(A)** Percentage of human CD45 in the blood of mice on study day 14 in the survival study. **(B)** Percentage and MFI of Granzyme in CD8 Treg in the blood (left) or splenocytes of mice terminated early due to clinical endpoints (right) in the MTX-101 dose titration study. **(C)** Serum concentrations of IFNγ in the survival study on day 14 (left) or with increasing dose of MTX-101on study day 42 (right) in mice with clinical disease scores 3 or greater. **(A–C)** Each symbol represents measurements from a single mouse. **(D)** H&E stain of disease affected intestinal tissue in anti-CD3 stimulated control (left) or MTX-101 (right) treated mice. Clinical disease scores of MTX-101 (green) relative to Saline (black) or abatacept technical control (red) groups (n=6/cohort). P values were determined by a unpaired t test. ns: p>0.05, *: p<0.05, **: p<0.01, ***: p<0.001, ****: p<0.0001.

Supplementary Figure 11 Additional donors/studies confirm 2mg/kg dose enhances survival via postulated MOA and support long term target engagement. **(A–D)** Small cohort survival study (n=4). Human NSG mice engrafted with 1x10^7^ human PBMC following irradiation (0.5 Gy) from donor LZ0007 were dosed with 2 mg/kg MTX-101 or saline intravenously every seven days from days 0-21. **(A)** Engraftment of human CD45 cells in the blood of saline and MTX-101 treated mice. **(B)** MTX-101 binding and MFI of Granzyme B on CD8 Treg on study day 14 in the blood, 2 hours post-dosing. Percentage of CD25 positive CD8 Treg in the blood on study day 14 (post-MTX-101 injections on study day 0 and 7) at 2 hours post-dose. **(C)** Percentage of Annexin V+ and CD25+ CD4 T cells on study day 14. **(D)** Survival curve for mice for small cohort survival study (n=4). Survival curves were compared using the Log-rank (Mantel-Cox) test. p=0.0069. **(E)** *In vivo* study experimental design. Human NSG mice were engrafted with 1x10^7^ human PBMC following irradiation (0.75 Gy) from donor AC3004 and dosed with 2mg/kg MTX-101 or saline every 7 days starting on day 0 out to study day 70. Each cohort consisted of 8 mice. **(F)** Human CD45 engraftment levels in the blood on study day 14. **(G)** MTX-101 binding to CD4, CD8, and CD8 Treg on study day 14, pre-dose in the blood. **(A–C, F, G)**. P values were determined by a unpaired t test. ns: p>0.05, *: p<0.05, **: p<0.01. **(H)** MTX-101 binding to CD4, CD8 and CD8 Treg in the blood on study day 49 and 70. Each symbol represents an individual mouse at that time point. **(H)** P values were determined by a one way ANOVA followed by by Šídák’s multiple comparisons test. **(A–E)** ns: p>0.05, *: p<0.05, **: p<0.01, ***: p<0.001.

Supplementary Figure 12 MTX-101 binds target cells in terminal tissues and does not result in the production of pro-inflammatory cytokines. **(A)** Percentage of T cells (CD3+) and NK cells (CD56+) in CD34+ NSG-Tg(Hu-IL-15) mice following 12 weeks of initial engraftment (n=24). P values were determined by an unpaired t test. ns: p>0.05, *: p<0.05, **: p<0.01. **(B)** Percentage of total Fc positive CD8 Treg, total CD8 T cells, or NK cells detected in the terminal spleens of MTX-101, vehicle, single arm anti-KIR, single arm anti-CD8, bivalent KIR antibody, or OKT3 (0.5 mg/kg) treated mice at the terminal time point (168 hrs). Each symbol in the graph represents a single mouse and each donor (n=2) is represented across 3 mice in each treatment group. P values were determined by a one-way ANOVA followed by by Šídák’s multiple comparisons test. **(A, B)** ns: p>0.05, *: p<0.05, **: p<0.01, ***: p<0.001, ****: p<0.0001. **(C)** Serum levels of proinflammatory cytokines for each of the treated mice in the study (n=6). Anti-CD3 (OKT3) is shown as a positive control for proinflammatory cytokine production.
