# Supplementary figures and images for: Preclinical characterization of MTX-101: a novel bispecific CD8 Treg modulator that restores CD8 Treg functions to suppress pathogenic T cells in autoimmune diseases
Source: Front Immunol. 2024 Nov 4;15:1452537. doi: 10.3389/fimmu.2024.1452537 (PMC11570885; doi:10.3389/fimmu.2024.1452537)

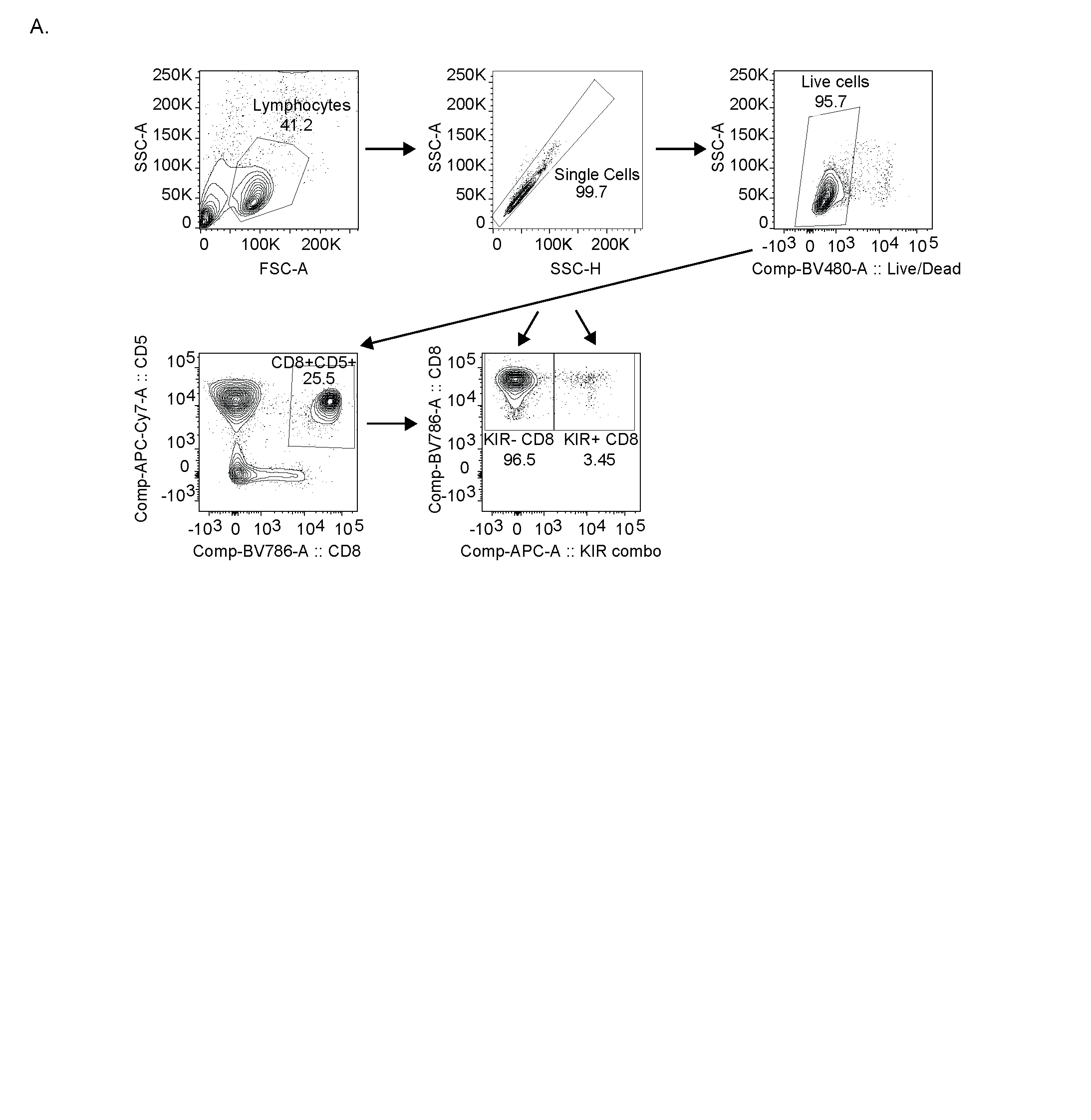

Supplement: Supplementary Figure 1 — Gating strategy for the identification of KIR+ CD8+ T cells. (A) Single lymphocytes were selected using SSC-A/FSC-A and SSC-A/SSC-H parameters respectively. Dead cells were excluded using LIVE/DEAD Fixable Aqua. CD8+ T cells were selected based on co-expression of CD8α and CD5. KIR+CD8+ and KIR-CD8+ T cells were identified phenotypically by co-staining for KIR antibodies. [file Image1.tif]

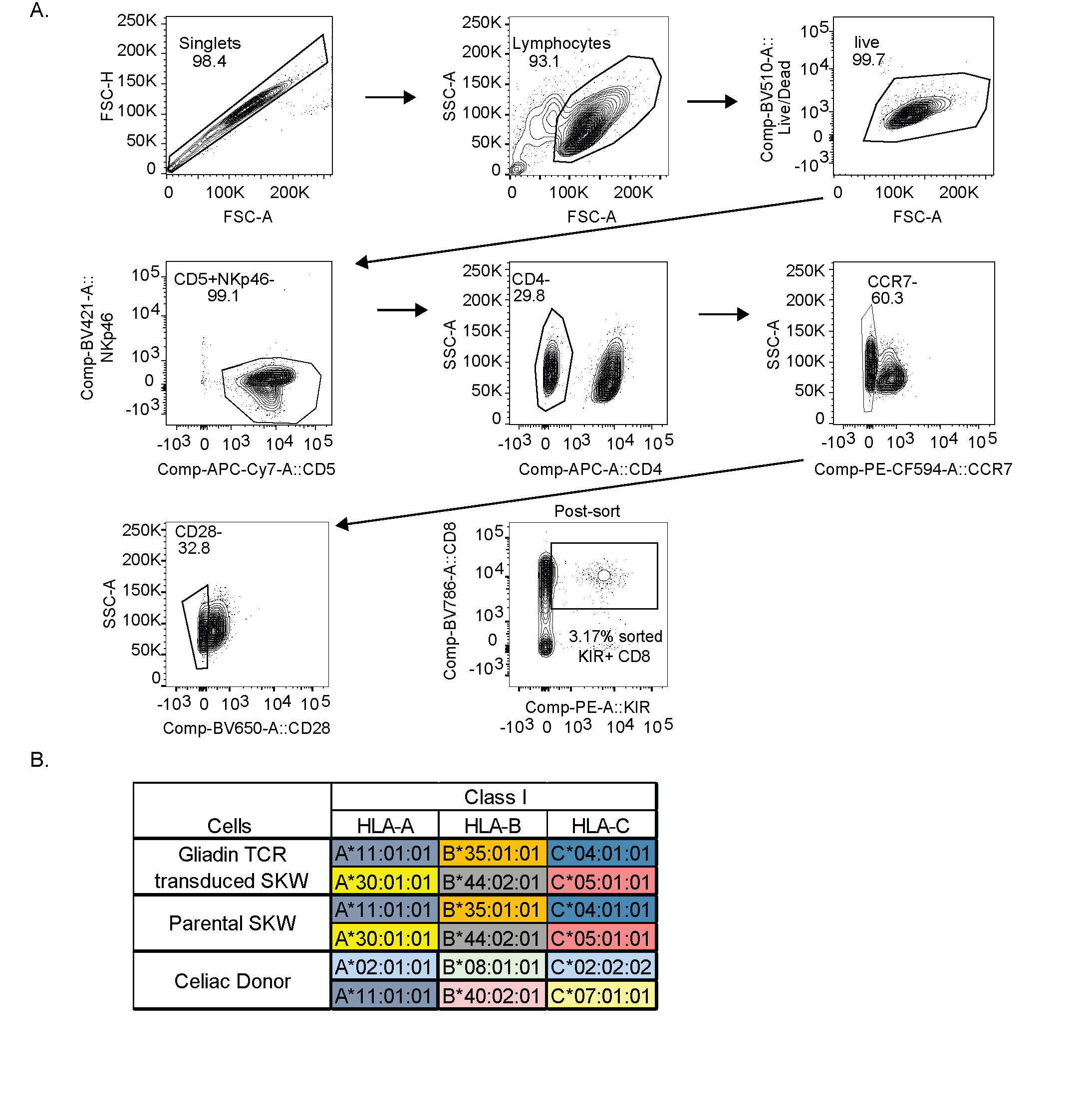

Supplement: Supplementary Figure 2 — Incucyte gating strategy and class I haplotype for CD8 Treg and gliadin TCR transduced and parental SKW target cells. (A) CD8 Treg enriched cells were sorted from celiac patient T cells following expansion in IL-7 and IL-15. Single cells were selected using FSC-H/FSC-A. Lymphocytes were selected using SSC-A/FSC-A. Dead cells were excluded using LIVE/DEAD Fixable Aqua. T cells were identified based on CD5 and NK cells were excluded based on NKp46. CD4 T cells were excluded. CCR7 and CD28 positive CD4- T cells were also eliminated to enrich for KIR+ CD8 T cells. The sorted population CD5+/NKp46-/CD4-/CCR7-/CD28- was then stained to determine %KIR+CD8. This percentage was then used to calculate total number of KIR+ CD8 T cells added to each incucyte well and CD8 Treg to target SKW ratios based on the initial number of SKW targets added to each well. (B) The MHC Class I haplotype of SKW target cells and celiac donor PBMCs used in the Incucyte assay. [file Image2.tif]

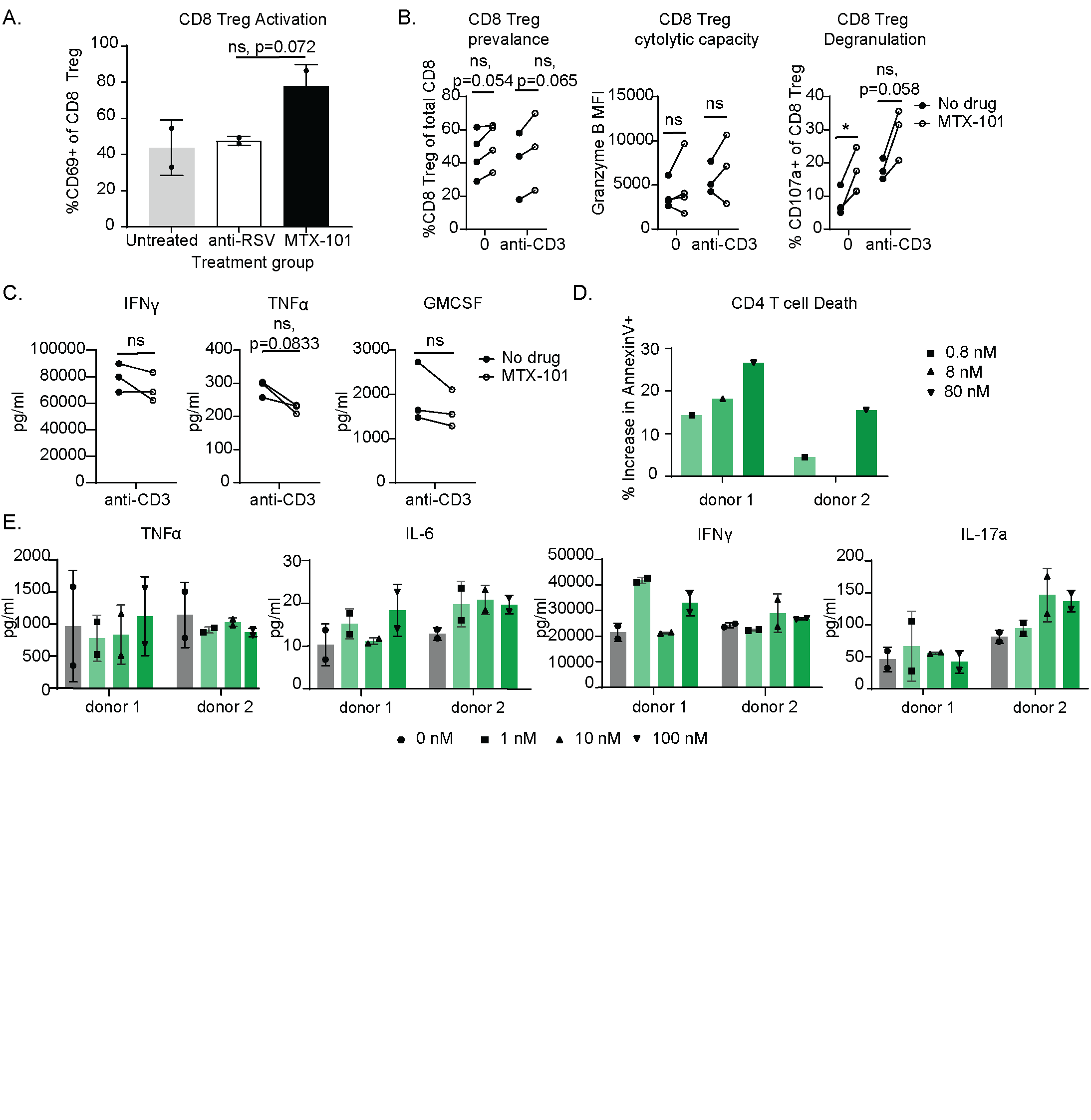

Supplement: Supplementary Figure 5 — CD8 Treg activation is not induced with control anti-RSV antibody and MTX-101 does not increase pro-inflammatory cytokine production in PBMC culture in the presence of anti-CD3 stimulation. (A) Percentage of CD69 positive CD8 Treg in healthy donor PBMC as detected by flow cytometry following incubation with no antibody, anti-RSV, or MTX-101 for 48 hours across two healthy donors. P values were determined by an unpaired t test. p=0.072, ns: p>0.05. (B) CD8 Treg prevalence, Granzyme B MFI, and degranulation quantified by CD107a expression following incubation of celiac donor CD8 Treg with CD4 target cells (1:5) and APCs in the presence or absence of 0.02 µg/ml anti-CD3 and MTX-101 (100nM) for three days. (C) IFNγ, TNFα, and GMCSF concentrations detected following the three day CD8 Treg and CD4 target co-culture as described in (B) in the presence of anti-CD3 with and without addition of MTX-101. (B, C) P values were determined by a paired t test. ns: p>0.05, *: p<0.05. (D) PBMC from two celiac donors were incubated with 0.8, 8 or, 80 nM of MTX-101 in the presence of low dose anti-CD3 (0.1 ug/mL) for 48 hours and endpoint %Annexin+ CD4s are shown. (E) Concentrations of the pro-inflammatory cytokines (TNFα, IL-6, IFNγ, and IL-17a) detected in the supernatant after incubation with low dose anti-CD3 in the presence or absence of MTX-101 for 48 hours across two different celiac donors. [file Image5.tif]

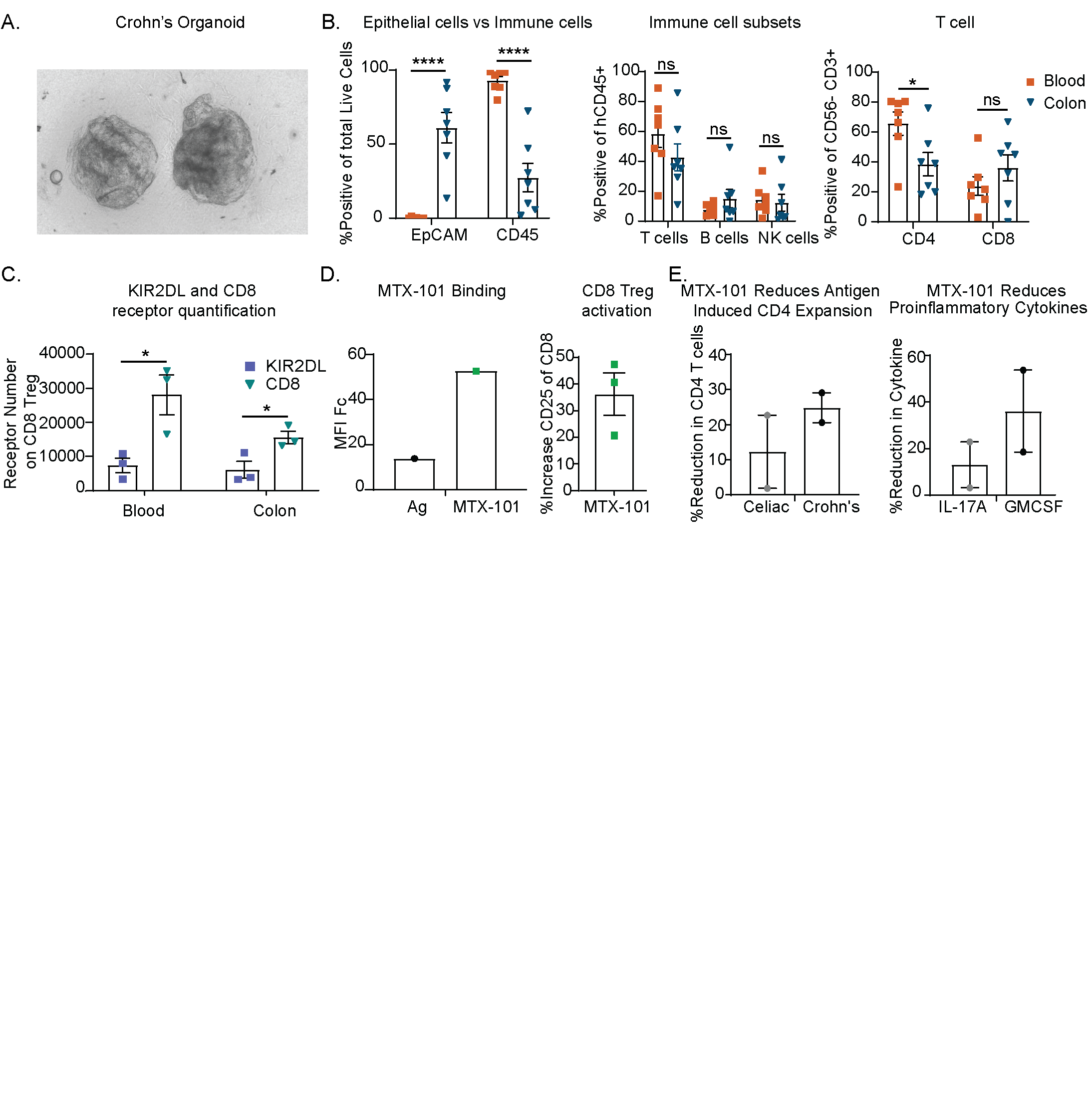

Supplement: Supplementary Figure 6 — Crohn’s Organoids expand from primary intestinal tissue samples and contain relevant immune cells. (A) Representative image of Crohn’s intestinal tissue derived organoids. (B) Percentage of epithelial (EpCAM+), T, B, and NK cells present in both blood and primary intestinal tissue (colon) of 7 different Crohn’s donors at baseline. T cell graph shows the percentage of CD4 and CD8 T cells detected in the blood and colon tissue at baseline. (C) The KIR2DL and CD8 receptor quantification per CD8 Treg in the blood and colon as determined by receptor MFI and quantitation beads. Results are representative of 3 independent Crohn’s donors. (B, C) P values were determined by a unpaired t test. ns: p>0.05, *: p<0.05, ** p<0.01, *** p<0.001, ****p<0.0001. (D) MTX-101 binding as detected with an anti-human IgG Fc antibody is shown for an MTX-101 treated well relative to an organoid well that did not receive MTX-101 (Ag), left. Percentage increase in CD8 Treg activation (CD25) as determined relative to the untreated control at study endpoint. n=3 independent experiments. (E) The reduction in antigen induced CD4 expansion following treatment with MTX-101 is shown across celiac and Crohn’s organoid cultures n=4 independent experiments (left). Percentage reduction in IL-17a and GMCSF detected in organoid supernatant following MTX-101 treatment n=2 celiac donors. [file Image6.tif]

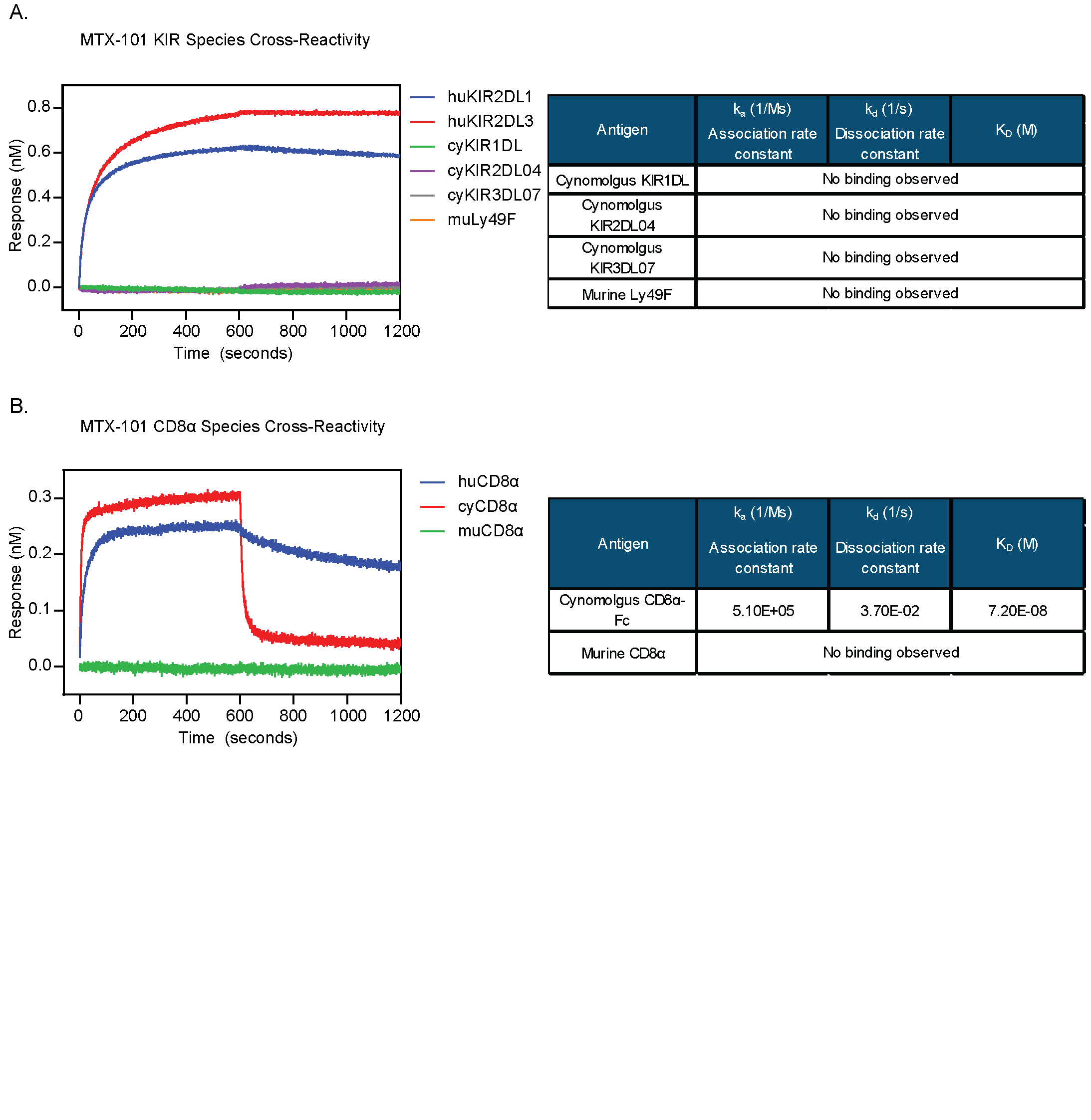

Supplement: Supplementary Figure 7 — MTX-101 lacks cross-reactivity to cyno KIR, murine Ly49F, and murine CD8α and shows some cross-reactivity to cyno CD8α. (A) Binding of 400 nM MTX-101 to KIR target antigens along with association and dissociation curves as detected by Bio-Layer Interferometry. (B) Binding of 400 nM MTX-101 to CD8α target antigens along with association and dissociation curves as detected by Bio-Layer Interferometry. (A, B) Tables show the association and dissociation rate constants measured for each binding interaction, and KD binding affinity calculated from the measured rate constants. [file Image7.tif]

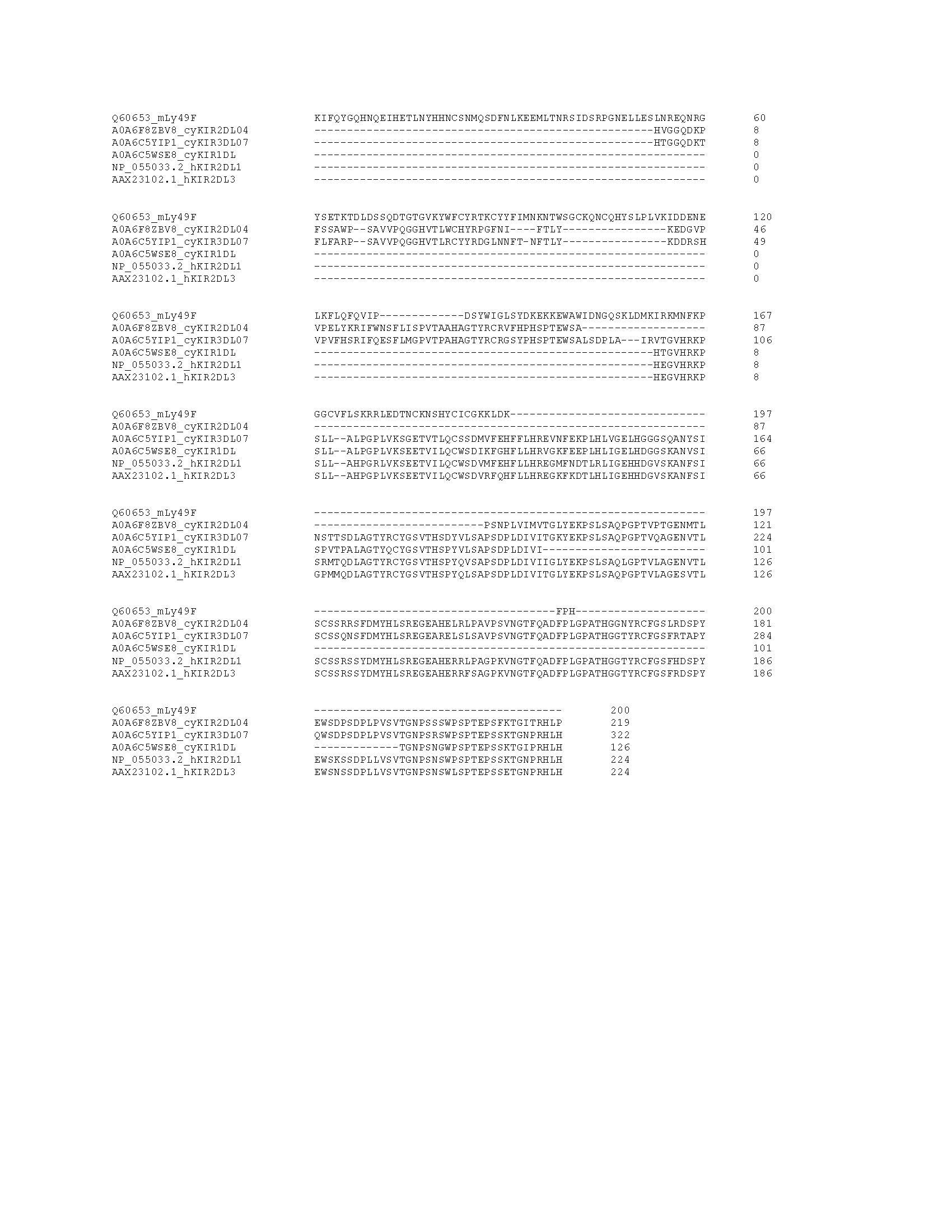

Supplement: Supplementary Figure 8 — Sequence alignment of the human and cyno KIR and mouse Ly49F extracellular domains generated by Clustal Omega. GenBank Accession numbers represent the sequence sources of either the commercially available hKIR or in-house generated cyKIR and mLy49F reagents used in the Octet cross-reactivity binding assay. A ‘-’ indicates a gap in the sequence alignment and a space indicates a non-conservative substitution. [file Image8.tif]

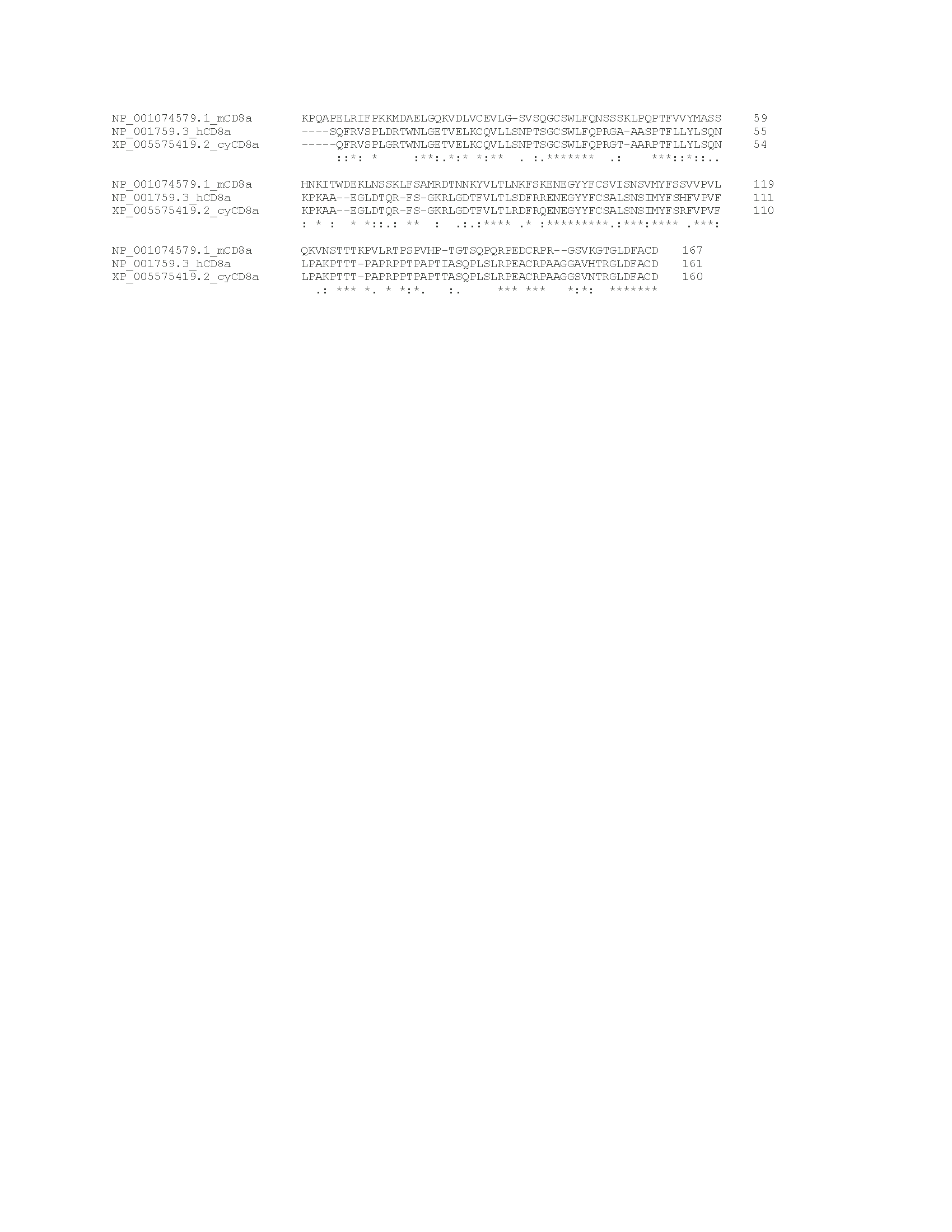

Supplement: Supplementary Figure 9 — Sequence alignment of the human, cyno and mouse CD8α extracellular domains generated by Clustal Omega. GenBank Accession numbers represent the sequence sources of the commercially available CD8α reagents used in the Octet cross-reactivity binding assay. A ‘-’ indicates a gap in the sequence alignment, a ‘*’ indicates the residue at that position is identical in all three sequences, a ‘:’ represents a conservative substitution, a ‘.’ represents a semi-conservative substitution at that position and a space indicates a non-conservative substitution. [file Image9.tif]

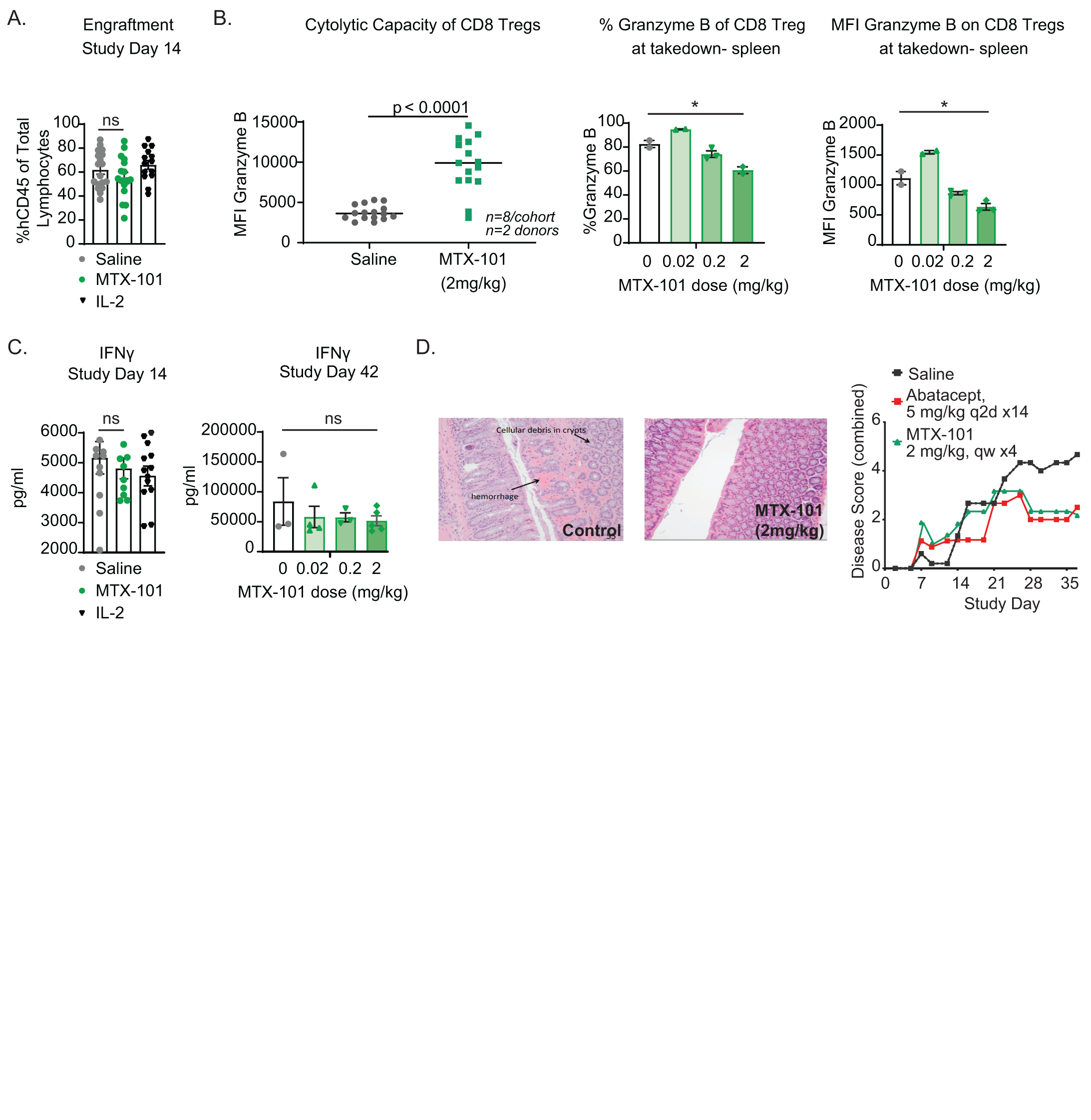

Supplement: Supplementary Figure 10 — Confirmation of human CD45 engraftment and dose-dependent decrease of Granzyme B in clinical endpoint mice. (A) Percentage of human CD45 in the blood of mice on study day 14 in the survival study. (B) Percentage and MFI of Granzyme in CD8 Treg in the blood (left) or splenocytes of mice terminated early due to clinical endpoints (right) in the MTX-101 dose titration study. (C) Serum concentrations of IFNγ in the survival study on day 14 (left) or with increasing dose of MTX-101on study day 42 (right) in mice with clinical disease scores 3 or greater. (A–C) Each symbol represents measurements from a single mouse. (D) H&E stain of disease affected intestinal tissue in anti-CD3 stimulated control (left) or MTX-101 (right) treated mice. Clinical disease scores of MTX-101 (green) relative to Saline (black) or abatacept technical control (red) groups (n=6/cohort). P values were determined by a unpaired t test. ns: p>0.05, *: p<0.05, **: p<0.01, ***: p<0.001, ****: p<0.0001. [file Image10.tif]

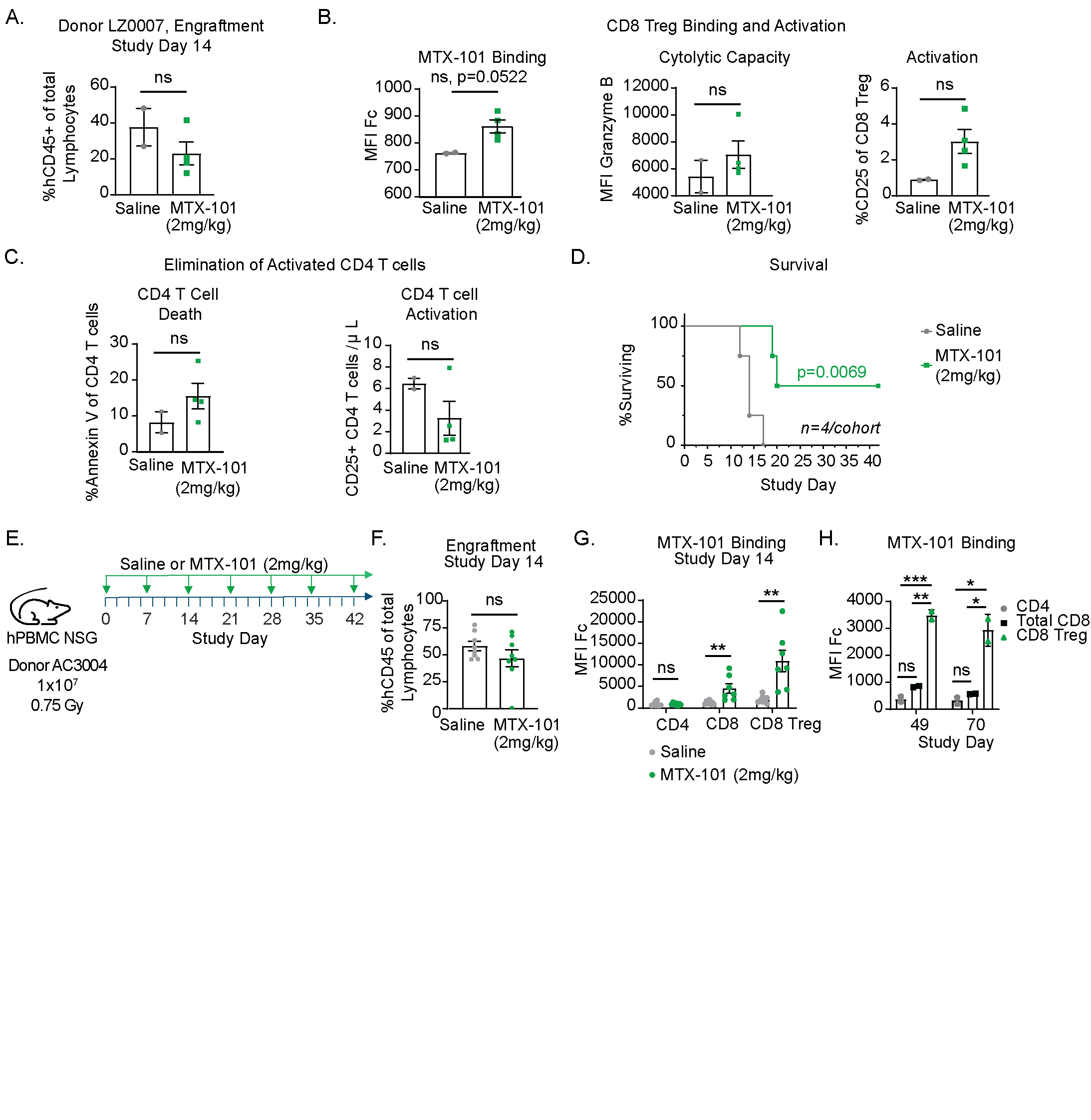

Supplement: Supplementary Figure 11 — Additional donors/studies confirm 2mg/kg dose enhances survival via postulated MOA and support long term target engagement. (A–D) Small cohort survival study (n=4). Human NSG mice engrafted with 1x10^7^ human PBMC following irradiation (0.5 Gy) from donor LZ0007 were dosed with 2 mg/kg MTX-101 or saline intravenously every seven days from days 0-21. (A) Engraftment of human CD45 cells in the blood of saline and MTX-101 treated mice. (B) MTX-101 binding and MFI of Granzyme B on CD8 Treg on study day 14 in the blood, 2 hours post-dosing. Percentage of CD25 positive CD8 Treg in the blood on study day 14 (post-MTX-101 injections on study day 0 and 7) at 2 hours post-dose. (C) Percentage of Annexin V+ and CD25+ CD4 T cells on study day 14. (D) Survival curve for mice for small cohort survival study (n=4). Survival curves were compared using the Log-rank (Mantel-Cox) test. p=0.0069. (E) In vivo study experimental design. Human NSG mice were engrafted with 1x10^7^ human PBMC following irradiation (0.75 Gy) from donor AC3004 and dosed with 2mg/kg MTX-101 or saline every 7 days starting on day 0 out to study day 70. Each cohort consisted of 8 mice. (F) Human CD45 engraftment levels in the blood on study day 14. (G) MTX-101 binding to CD4, CD8, and CD8 Treg on study day 14, pre-dose in the blood. (A–C, F, G). P values were determined by a unpaired t test. ns: p>0.05, *: p<0.05, **: p<0.01. (H) MTX-101 binding to CD4, CD8 and CD8 Treg in the blood on study day 49 and 70. Each symbol represents an individual mouse at that time point. (H) P values were determined by a one way ANOVA followed by by Šídák’s multiple comparisons test. (A–E) ns: p>0.05, *: p<0.05, **: p<0.01, ***: p<0.001. [file Image11.tif]

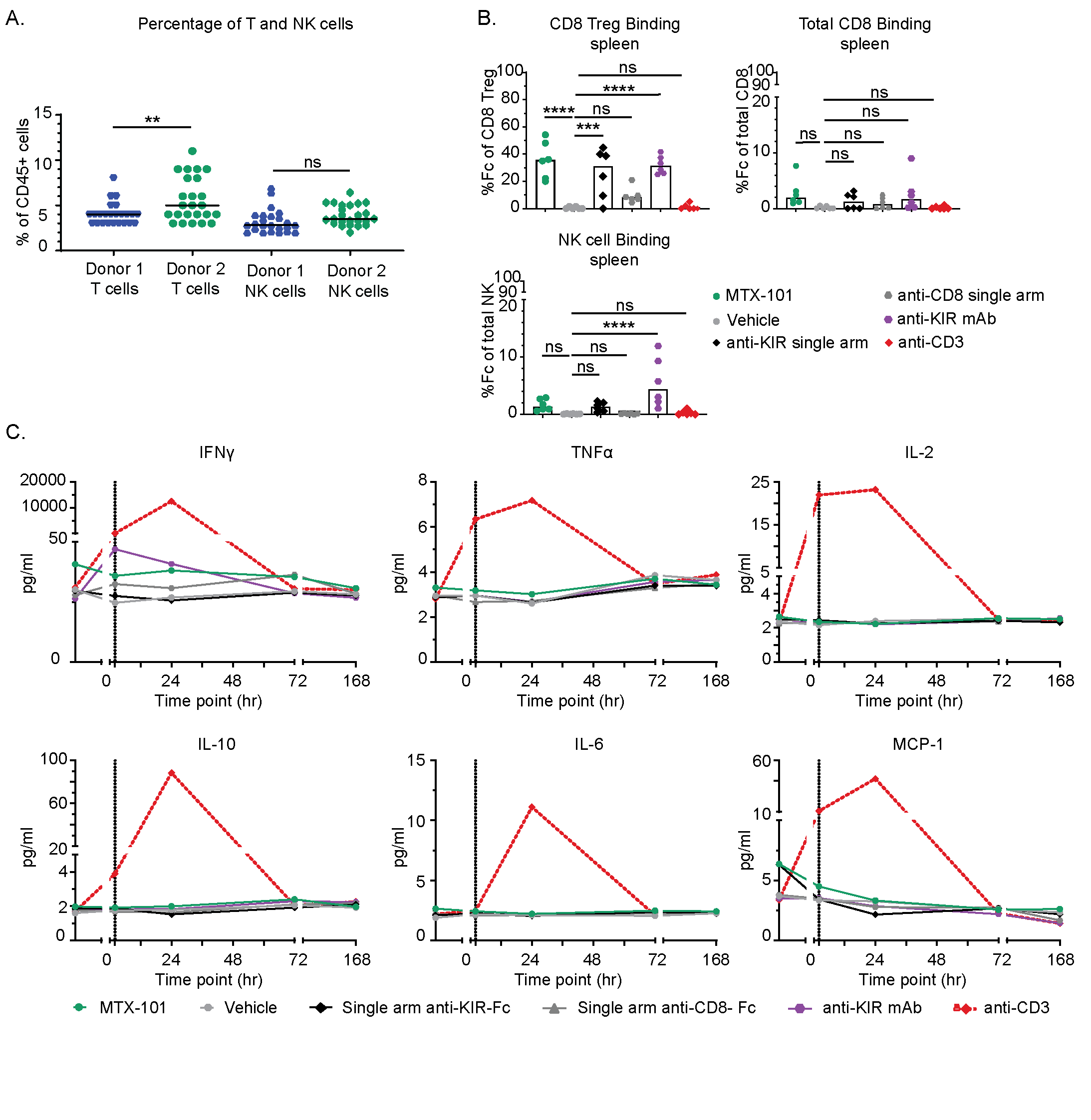

Supplement: Supplementary Figure 12 — MTX-101 binds target cells in terminal tissues and does not result in the production of pro-inflammatory cytokines. (A) Percentage of T cells (CD3+) and NK cells (CD56+) in CD34+ NSG-Tg(Hu-IL-15) mice following 12 weeks of initial engraftment (n=24). P values were determined by an unpaired t test. ns: p>0.05, *: p<0.05, **: p<0.01. (B) Percentage of total Fc positive CD8 Treg, total CD8 T cells, or NK cells detected in the terminal spleens of MTX-101, vehicle, single arm anti-KIR, single arm anti-CD8, bivalent KIR antibody, or OKT3 (0.5 mg/kg) treated mice at the terminal time point (168 hrs). Each symbol in the graph represents a single mouse and each donor (n=2) is represented across 3 mice in each treatment group. P values were determined by a one-way ANOVA followed by by Šídák’s multiple comparisons test. (A, B) ns: p>0.05, *: p<0.05, **: p<0.01, ***: p<0.001, ****: p<0.0001. (C) Serum levels of proinflammatory cytokines for each of the treated mice in the study (n=6). Anti-CD3 (OKT3) is shown as a positive control for proinflammatory cytokine production. [file Image12.tif]
