# Supplementary material for: Preclinical characterization of MTX-101: a novel bispecific CD8 Treg modulator that restores CD8 Treg functions to suppress pathogenic T cells in autoimmune diseases
Source: Front Immunol. 2024 Nov 4;15:1452537. doi: 10.3389/fimmu.2024.1452537 (PMC11570885; doi:10.3389/fimmu.2024.1452537)
Supplement: Supplementary Figure 3 — CD8 T cells enriched for Treg eliminate activated gliadin-responsive CD4 targets. (A–C) CD8 T cells were enriched from celiac donor PBMC following expansion in IL-7 and IL-15 for seven days and sorted on the surface markers KLRG1+CD244+CD28- (CD8 Treg enriched effectors) or KLRG1-CD244-CD28+ (non-CD8 Treg effectors). CD8 enriched effectors were combined with activated gliadin-responsive GFP+ SKW CD4 target cell line at a 2 to 1 ratio. Percent change in GFP+ objects from an 8 hour time point is shown for both CD8 Treg and non-CD8 Treg effectors over 48 hours cultured with targets. Activated targets only in the absence of addition of CD8 effectors are shown as a representative control. (B, C) Incucyte-generated images show the decrease in GFP+ gliadin-responsive SKW target cells over 48 hours for both CD8 Treg enriched (B) and Non-CD8 Treg effector cells (C). [file Image3.pdf]

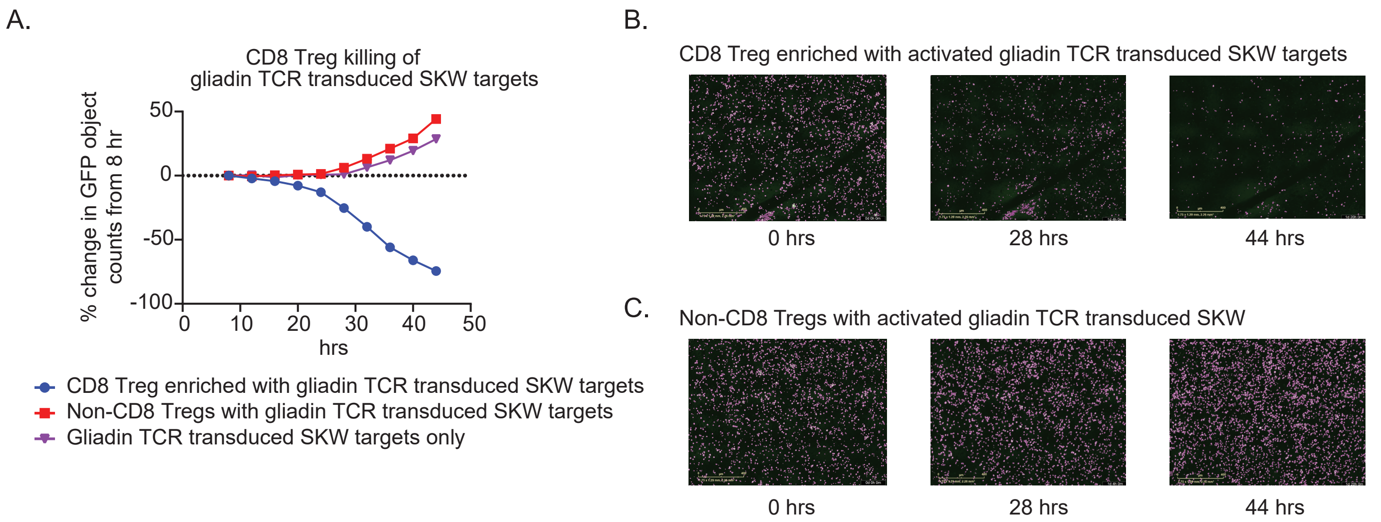
A.
CD8 Treg killing of
gliadin TCR transduced SKW targets
% change in GFP object counts from 8 hr
50
0
-50
-100
0
10
20
30
40
50
hrs
CD8 Treg enriched with gliadin TCR transduced SKW targets
Non-CD8 Tregs with gliadin TCR transduced SKW targets
Gliadin TCR transduced SKW targets only
B.
CD8 Treg enriched with activated gliadin TCR transduced SKW targets
0 hrs
28 hrs
44 hrs
C.
Non-CD8 Tregs with activated gliadin TCR transduced SKW
0 hrs
28 hrs
44 hrs
