# Supplementary material for: Preclinical characterization of MTX-101: a novel bispecific CD8 Treg modulator that restores CD8 Treg functions to suppress pathogenic T cells in autoimmune diseases
Source: Front Immunol. 2024 Nov 4;15:1452537. doi: 10.3389/fimmu.2024.1452537 (PMC11570885; doi:10.3389/fimmu.2024.1452537)
Supplement: Supplementary Figure 4 — MTX-101 binding is dose-dependent and specific to target proteins. (A) Dose-dependent binding curves for MTX-101 to KIR2DL1/2/3 and CD8α target antigens along with association and dissociation curves as detected by Bio-Layer Interferometry. (B) MTX-101 binding to KIR3DL1 transfected HEK 293 or untransfected cells. Dose-dependent binding of a KIR3DL1 targeted bispecific antibody is shown as a positive control on the KIR3DL1 transfected cell line. (C) Retrogenix live cell microarray technology study evaluated surface protein library transfected 293T cell binding of MTX-101 and single arm controls. Summary table of target binding is shown. Each KIR isoform tested and binding to MTX-101 or single arm controls (KIR Fab or CD8 scFv) is shown for both fixed and unfixed samples. Binding summary of CD8α is also shown for MTX-101 and controls. Of the 6,500 proteins tested there was also weak binding to Frizzled Class Receptor 5 (FZD5) as shown in the table. Strong and medium binding is coded as green in the table, weak binding is coded as blue, very (v.) weak binding is yellow, and no binding (neg) is white. [file Image4.pdf]

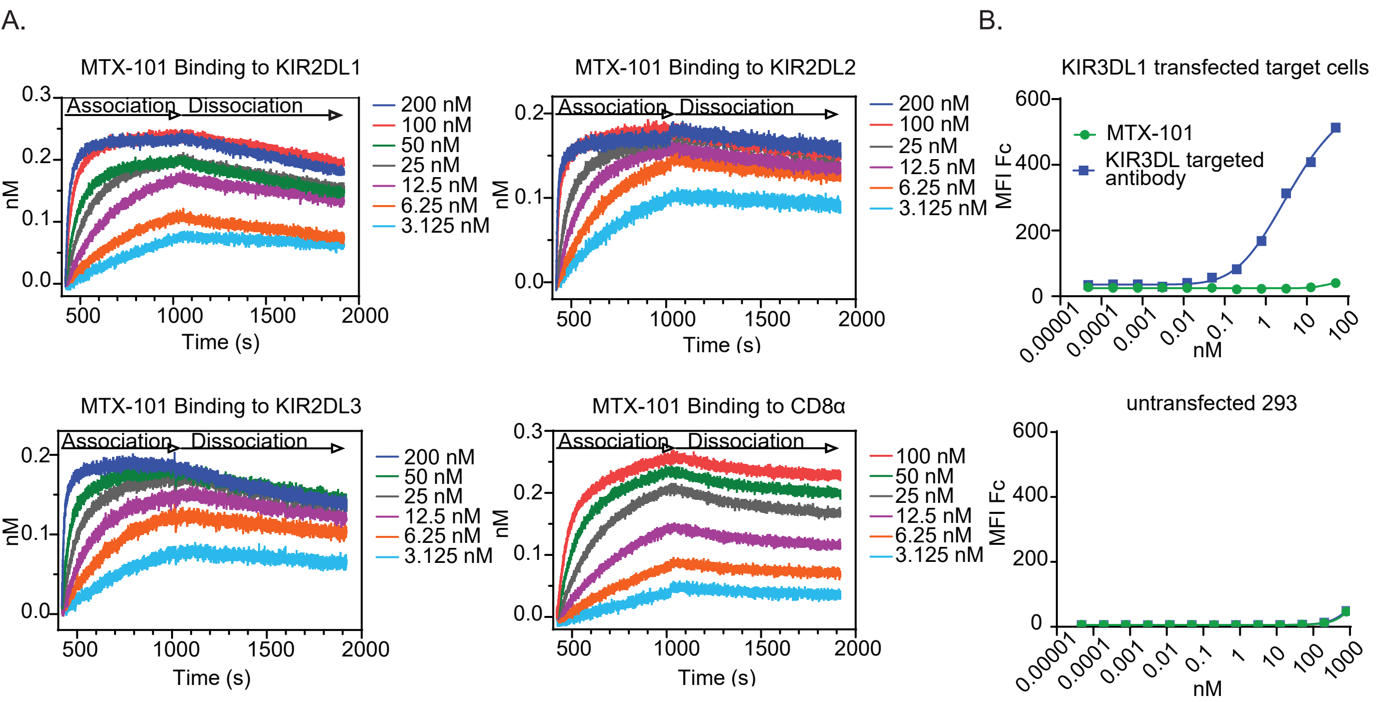

C.

Screen Results- fixed	KIR2DS2	KIR2DS3	KIR2DS1	KIR2DL2	KIR2DL3	KIR2DL1	KIR2DS2	KIR2DL1	CD8A	CD8A	FZD5
Sample ID (Concentration)	246	1006	1058	927	2240	1942	3468	3402	4948	5802	1819
MTX-101 (20 µg/mL)	med/strong	weak	strong	strong	strong	strong	strong	strong	med/strong	medium	v weak
MTX-101 (20 µg/mL)	med/strong	weak	strong	strong	strong	strong	strong	strong	medium	medium	v weak
KIR FAB Fc (20 µg/mL)	med/strong	weak	strong	strong	strong	strong	strong	strong	neg	neg	v weak
KIR FAB Fc (20 µg/mL)	med/strong	weak	strong	strong	strong	strong	strong	strong	neg	neg	v weak
CD8 scFv Fc (20 µg/mL)	neg	neg	neg	neg	neg	neg	neg	neg	medium	weak	neg
CD8 scFv Fc (20 µg/mL)	neg	neg	neg	neg	neg	neg	neg	neg	medium	weak/med	neg
Screen Results- unfixed	KIR2DS2	KIR2DS3	KIR2DS1	KIR2DL2	KIR2DL3	KIR2DL1	KIR2DS2	KIR2DL1	CD8A	CD8A	FZD5
MTX-101 (20 µg/mL)	weak/med	v weak	medium	medium	medium	weak/med	medium	strong	med/strong	v weak	weak
KIR FAB Fc (20 µg/mL)	weak/med	v weak	weak/med	medium	weak/med	weak	medium	strong	neg	neg	weak
CD8 scFv Fc (20 µg/mL)	neg	neg	neg	neg	neg	neg	neg	neg	med/strong	v weak	neg
