# Supplementary material for: Preclinical characterization of MTX-101: a novel bispecific CD8 Treg modulator that restores CD8 Treg functions to suppress pathogenic T cells in autoimmune diseases
Source: Front Immunol. 2024 Nov 4;15:1452537. doi: 10.3389/fimmu.2024.1452537 (PMC11570885; doi:10.3389/fimmu.2024.1452537)
Supplement: Supplementary file 13 [file Table1.docx]

S. Table 1. Celiac Peptides Sequence Information

| Name | Supplier | Peptide Sequence |
| --- | --- | --- |
| Gliadin peptide NPL001 (Protein: α-gliadin) | Elim Biopharm | (pE)LQPFPQPELPYPQPQ-NH2 |
| Gliadin peptide NPL002 (Protein: ω-gliadin/hordein) | Elim Biopharm | (pE)QPFPQPEQPFPWQP-NH2 |
| Gliadin peptide NPL003 (Protein: hordein) | Elim Biopharm | (pE)PEQPIPEQPQPYPQQ-NH2 |
| DQ2.5-glia-α1a peptide | Elim Biopharm | QLQPFPQPELPY |
| DQ2.5-glia-α2 peptide | Elim Biopharm | PQPELPYPQPE |
| DQ2.5-glia-ω1 peptide | Elim Biopharm | QQPFPQPEQPFP |
| DQ2.5-glia-ω2 peptide | Elim Biopharm | FPQPEQPFPWQP |
