# Supplementary material for: Preclinical characterization of MTX-101: a novel bispecific CD8 Treg modulator that restores CD8 Treg functions to suppress pathogenic T cells in autoimmune diseases
Source: Front Immunol. 2024 Nov 4;15:1452537. doi: 10.3389/fimmu.2024.1452537 (PMC11570885; doi:10.3389/fimmu.2024.1452537)
Supplement: Supplementary file 14 [file Table2.docx]

Raw Celiac Data

| Readout | Celiac Peptides | Peptides+MTX-101 | p value |
| --- | --- | --- | --- |
| CD8 Treg | **37.95** | **44.35** | 0.0994 |
|  | **31.15** | **36.30** |  |
|  | 64.90 | 64.10 |  |
|  | **52.60** | **61.90** |  |
| Granzyme B MFI | **13603.00** | **18950.50** | 0.3487 |
|  | 2787.50 | 1588.00 |  |
|  | **4531.50** | **5555.50** |  |
|  | **4381.50** | **5299.50** |  |
| CD107a | **23.45** | **36.75** | 0.0704 |
|  | **6.06** | **16.95** |  |
|  | **7.73** | **14.85** |  |
|  | **12.20** | **12.40** |  |
|  |  |  |  |
| IFNγ | **21756.23** | **14902.35** | 0.1591 |
|  | **74707.98** | **63156.82** |  |
| TNFα | **329.42** | **294.11** | 0.0957 |
|  | **263.90** | **216.02** |  |
| GMCSF | **1201.91** | **932.45** | * 0.0145 |
|  | **3037.35** | **2755.35** |  |

S. Table 2. Raw flow data and cytokine data for Figure 3. The percentage of CD8 Tregs is shown for each of the donors tested in CD8 Treg co-cultures with and without the addition of MTX-101 for 3 days as described in Figure 3. Granzyme B MFI and the percentage of CD107 positive CD8 Tregs is also shown for each of the donors tested in the assay following addition of MTX-101. Supernatants from co-cultures were taken at the end of the assay and value of the concentration (pg/mL) of each proinflammatory cytokine is shown. P values indicated results from a paired t-test for each parameter across donors.
