# Supplementary material for: Preclinical characterization of MTX-101: a novel bispecific CD8 Treg modulator that restores CD8 Treg functions to suppress pathogenic T cells in autoimmune diseases
Source: Front Immunol. 2024 Nov 4;15:1452537. doi: 10.3389/fimmu.2024.1452537 (PMC11570885; doi:10.3389/fimmu.2024.1452537)
Supplement: Supplementary file 15 [file Table3.docx]

Raw Crohn’s and Organoid Data

| Readout | Indication | Antigen | Ag+MTX-101 | p value |
| --- | --- | --- | --- | --- |
| Granzyme | Crohn's Donor | 3131 | 2052 | 0.1019 |
|  | Crohn's Donor | **322** | **716** |  |
|  | Crohn's Donor | **1286** | **1819** |  |
|  | Crohn's Donor | **3486** | **3964** |  |
|  | Crohn's Donor | **12226** | **13234** |  |
|  | Crohn's Donor | 12782 | 12439 |  |
|  | Crohn's Donor | **11426** | **12954** |  |
|  | Crohn's Donor | **2805** | **7249** |  |
|  | Crohn's Donor | **4290** | **5405** |  |
|  | Crohn's Donor | **11617** | **11973** |  |
| Proliferation due to Ag stimulation | Crohn's Donor | **33.9** | **27.2** | 0.523 |
|  | Crohn's Donor | **9.04** | **8.61** |  |
|  | Crohn's Donor | **21.8** | **16.1** |  |
|  | Crohn's Donor | 32.5 | 32.3 |  |
|  | Crohn's Donor | 30.7 | 31.5 |  |
|  | Crohn's Donor | **38** | **29.3** |  |
|  | Crohn's Donor | 11.4 | 12.3 |  |
|  | Crohn's Donor | 18.8 | 27.7 |  |
| IFNγ | Crohn's Donor | **21452.33** | **6864.626** | 0.2736 |
|  | Crohn's Donor | **3945.48** | **2254.108** |  |
|  | Crohn's Donor | **28106.95** | **15750.29** |  |
|  | Crohn's Donor | **19679.38** | **16120.3** |  |
|  | Crohn's Donor | **20170.01** | **19211.94** |  |
|  | Crohn's Donor | **35221.07** | **15758.28** |  |
|  | Crohn's Donor | 136088.3 | 138037.4 |  |
|  | Crohn's Donor | 43753.9 | 58253.18 |  |
| TNFα | Crohn's Donor | **180.2901** | **103.4044** | *  0.0498 |
|  | Crohn's Donor | **3.676221** | **3.25557** |  |
|  | Crohn's Donor | **273.0472** | **167.8592** |  |
|  | Crohn's Donor | 69.38083 | 79.91141 |  |
|  | Crohn's Donor | **55.74198** | **41.36979** |  |
|  | Crohn's Donor | **137.1445** | **64.92977** |  |
|  | Crohn's Donor | **377.4113** | **344.0726** |  |
|  | Crohn's Donor | 46.81791 | 48.85386 |  |
| Epithelial Cell Death in Organoids | Celiac | **83.6** | **72** | *  0.0176 |
|  | Celiac | **80.3** | **70.3** |  |
|  | Celiac | **92.2** | **90.6** |  |
|  | Crohn's | **83.7** | **78.5** |  |
|  | Crohn's | **79** | **66** |  |

S. Table 3. Raw flow data and cytokine data for Figure 4. For Granzyme, IFNγ, and TNFα the concentration of cytokine detected in the media following stimulation with the Crohn’s antigen mix as specified for Figure 4. For epithelial cell death, the percentage dead epithelial cells as detected by a viability dye and flow cytometry is shown in the presence and absence of MTX-101. Proliferation refers to the percentage of CD4 T cells that have fully diluted CFSE due to antigenic stimulation by the end of the seven-day assay. P values indicated results from a paired t-test for each parameter across donors.
