# Supplementary material for: Preclinical characterization of MTX-101: a novel bispecific CD8 Treg modulator that restores CD8 Treg functions to suppress pathogenic T cells in autoimmune diseases
Source: Front Immunol. 2024 Nov 4;15:1452537. doi: 10.3389/fimmu.2024.1452537 (PMC11570885; doi:10.3389/fimmu.2024.1452537)
Supplement: Supplementary file 16 [file Table4.docx]

**S. Table 4.** Percent (%) sequence identity between the human and cyno KIR and mouse Ly49F extracellular domains. GenBank Accession numbers represent the sequence sources of the commercially available hKIR or in-house generated cyKIR and mLy49F reagents used in the Octet cross-reactivity binding assay and the multiple sequence alignment. The cyKIR alleles analyzed herein were selected because they contained the highest percent sequence identity to hKIR2DL1, hKIR2DL3 and hKIR2DL3 canonical sequences.

| KIR species | hKIR2DL1 | hKIR2DL3 | cyKIR1DL | cyKIR2DL04 | cyKIR3DL07 | mLy49F |
| --- | --- | --- | --- | --- | --- | --- |
| NP_055033.2_hKIR2DL1 | 100.00 |  |  |  |  |  |
| AAX23102.1_hKIR2DL3 | 91.52 | 100.00 |  |  |  |  |
| A0A6C5WSE8_cyKIR1DL | 80.95 | 81.75 | 100.00 |  |  |  |
| A0A6F8ZBV8_cyKIR2DL04 | 81.06 | 79.55 | 73.53 | 100.00 |  |  |
| A0A6C5YIP1_cyKIR3DL07 | 77.68 | 78.12 | 79.37 | 77.63 | 100.00 |  |
| Q60653_mLy49F | 17.95 | 15.38 | 13.89 | 18.18 | 16.13 | 100.00 |
