# Supplementary material for: Preclinical characterization of MTX-101: a novel bispecific CD8 Treg modulator that restores CD8 Treg functions to suppress pathogenic T cells in autoimmune diseases
Source: Front Immunol. 2024 Nov 4;15:1452537. doi: 10.3389/fimmu.2024.1452537 (PMC11570885; doi:10.3389/fimmu.2024.1452537)
Supplement: Supplementary file 17 [file Table5.docx]

**S. Table 5.** Percent (%) sequence identity between the human, cyno and mouse CD8α extracellular domains. GenBank Accession numbers represent the sequence sources of the commercially available CD8α reagents used in the Octet cross-reactivity binding assay and the multiple sequence alignment.

| CD8 species | hCD8α | cyCD8α | mCD8α |
| --- | --- | --- | --- |
| NP_001759.3_hCD8α | 100.00 |  |  |
| XP_005575419.2_cyCD8α | 94.38 | 100.00 |  |
| NP_001074579.1_mCD8α | 45.22 | 46.15 | 100.00 |
